# Publisher Correction: Proton mediated spin state transition of cobalt heme analogs

**DOI:** 10.1038/s41467-019-10880-z

**Published:** 2019-06-21

**Authors:** Jianping Zhao, Qian Peng, Zijian Wang, Wei Xu, Hongyan Xiao, Qi Wu, Hao-Ling Sun, Fang Ma, Jiyong Zhao, Cheng-Jun Sun, Jianzhang Zhao, Jianfeng Li

**Affiliations:** 1 College of Materials Science and Opto-electronic Technology, CAS Center for Excellence in Topological Quantum Computation, & Center of Materials Science and Optoelectronics Engineering, University of Chinese Academy of Sciences, Yanqi Lake, Huairou District, 101408 Beijing, China; 2 State Key Laboratory and Institute of Elemento-Organic Chemistry, College of Chemistry, Nankai University, 300071 Tianjin, China; 3 Institute of High Energy Physics & University of Chinese Academy of Sciences, Chinese Academy of Sciences, 100049 Beijing, China; 4 Key Laboratory of Photochemical Conversion and Optoelectronic Materials, Technical Institute of Physics and Chemistry, Chinese Academy of Sciences, 100190 Beijing, China; 5 Department of Chemistry and Beijing Key Laboratory of Energy Conversion and Storage Materials, Beijing Normal University, 100875 Beijing, China; 6 Advanced Photon Source, Argonne National Laboratory, Argonne, IL 60439 USA; 7 State Key Laboratory of Fine Chemicals, School of Chemical Engineering, Dalian University of Technology, West Campus, 2 Ling-Gong Road, 116024 Dalian, China

**Keywords:** Metalloproteins, Chemical bonding

Correction to: *Nature Communications* 10.1038/s41467-019-10357-z, published online 24 May 2019.

In the original version of this Article, the four structural depictions of orbital distributions were inadvertently omitted from Fig. 5. This has now been corrected in both the HTML and PDF versions of the Article.

