# Supplementary material for: Proton mediated spin state transition of cobalt heme analogs
Source: Nat Commun. 2019 May 24;10:2303. doi: 10.1038/s41467-019-10357-z (PMC6534676; doi:10.1038/s41467-019-10357-z)
Supplement: Supplementary file 1 — Supplementary Information [file 41467_2019_10357_MOESM1_ESM.pdf]

Supplementary Information

Proton mediated spin state transition of cobalt heme analogues

Zhao et al.

Supplementary Methods

General Information. All reactions and manipulations were carried out under argon using a double-manifold vacuum line, Schlenkware, and cannula techniques unless otherwise noted. Tetrahydrofuran (THF) was distilled over sodium/benzophenone, chlorobenzene over P_2O_5, hexane over potassium-sodium alloy, and heptane over sodium under nitrogen. KH (aladdin chemicals) was stored in the dry-box and washed with hexanes before use. Kryptofix 222 (ACROS) was purified by vacuum sublimation. 2-methylimidazole was recrystallized from benzene and dried under vacuum. Potassium 2-methylimidazolate ([K(2-MeIm^{-})]) was prepared according to Hu et al.[1] [H_2(TPP)] and [H_2(TTP)] were prepared according to Adler et al.[2] The metalation of the free-base porphyrins to give [Co(Porph)] (Porph = porphyrin) was done by a modification of a literature method.[3] UV-vis (THF) of [Co(TPP)] λmax: 415, 528 nm and UV-vis (THF) of [Co(TTP)] λmax: 416, 528 nm. UV-vis spectra were recorded on a PerkinElmer Lambda 25 UV-vis Spectrometer.

EPR measurements and simulations: EPR were carried out on a Bruker EMX plus 10/12 CW X-band ESR/EPR spectrometer, equipped with High-Q cylindrical cavity and Oxford ESR910 continuous flow liquid helium cryostat. The EPR spectra were measured at different temperatures between 2 and 90 K with a modulation amplitude 0.2 mT, a modulation frequency of 100 kHz, and a microwave power of 10.02 mW. The EPR spectra were simulated and fitted to the experimental data using EasySpin,[4] which is operated in MATLAB.

[Co(TPP)(2-MeHIm)] Simulation parameters: *S* = 1/2; g = [2.32, 2.0]; A(^{59}Co) = -[5, 79]*2.8 MHz; lwpp = 6 linewidth mT.

[Co(TPP)(2-MeIm^{-})]$^{-}$ Simulation parameters: *S* = 3/2; g = [3.0, 1.95, 2.01]; A(^{59}Co) = -[1, 82]*2.8 MHz; lwpp = 6 linewidth mT; D = -1.667 cm^{-1}; E/D = 0. HStrain = [0.1 0.1 0.1]; gStrain = [0.28, 0.21, 0.001].

Magnetometry: Variable-temperature magnetic susceptibility measurements were performed on Quantum Design SQUID-MPMS3 (1~1000 Hz) magnetometer. The experimental susceptibilities were corrected for the diamagnetism of the constituent atoms (Pascal's tables) and background of the sample holder.

The X-ray Absorption Fine structure spectroscopy (XAFS) : data were recorded at beamline 20 BM of Advanced Photon Source at Argonne National Laboratory, using the Si(111) double crystal monochromator to scan the energy. The spectra were collected in transmission mode and energy calibration were done using Co foil as references. The experimental XANES spectra at Co *K*-edge were normalized to +100eV post the absorption edge after subtracting the atomic absorption background using the Demeter package.[5] Theoretical Co K-edge XANES spectra as well as projected partial density of states were calculated using the Full Multiple Scattering Theory with muffin-tin approximation, as implemented in FEFF9.6 code.[6] A Hedin-Lundqvist correlation potential was adopted for the atomic scattering potential. The cluster radius around central Co for self-consistent field (SCF) and full multiple scattering (FMS) calculations are 5 and 10 Å, respectively. The Fermi energy was shifted by 3 eV to allow the appearance of deep level d states and additional broadening of 1 eV was applied to account for the instrumental resolution. The crystal models were taken from the refined XRD structures.

Electronic Structure: The G09 program package[7] was used to optimize the structures and for frequency analysis in our study. The model complex for [Co(TPP)(2-MeIm^{-})]$^{-}$ and [Co(TPP)(2-MeHIm)] were fully optimized without any constraints by using the Hybrid-GGA functional U-M06[8] and U-B3LYP[9,10].The triple-ζ valence basis sets TZVP[11] was used for Co, and 6-31+G* basis sets for N, 6-31G* basis set for all other atoms. Two different spin states low-spin (S = 1/2) or high spin (S = 3/2) of model complex were calculated in order to estimate ground states. All optimized species were verified minima structures by the presence of zero imaginary vibrational frequency. Free energies were evaluated at 298K and 1atm using harmonic vibrational frequencies at the same basis level.

Synthesis of [K(2-MeIm^{-})]: [K(2-MeIm^{-})] was synthesized by reaction of 2-MeHIm with less than 1 equiv of KH in dry-box. Typically, 0.132 g of KH (3.30 mmol) was dissolved in 20 mL of THF, and 0.3 g of 2-MeHIm (3.66 mmol) was added over half hours. The resulting mixture was stirred 2 hours and the product was isolated by filtration, washed with THF (3×15 mL), and then dried under vacuum for 2 hours.

Synthesis of [Co(TPP)(2-MeHIm)]: [Co(TPP)] (10 mg, 0.015 mmol) was dried in vacuum for 1 h. Excess 2-methylimidazole (12 mg, 0.146 mmol) in THF (5 mL) was added to the [Co(TPP)] solid by cannula. The mixture was stirred for 30 min and transferred into glass tubes (8 mm × 250 mm) which were layered with hexanes as the nonsolvent. X-ray quality crystals were collected after 1 week.

Synthesis of [Co(TTP)(2-MeHIm)]: [Co(TTP)] (10 mg, 0.014 mmol) was dried in vacuum for 1 h and dissolved in chlorobenzene (5 mL). This solution was transferred to a Schlenk flask containing excess 2-methylimidazole (12 mg, 0.146 mmol) via cannula, after stirring for 1 h and the solution was filtered. X-ray quality crystals were obtained after 2 weeks by liquid diffusion using hexanes as nonsolvent in glass tube (8 mm × 250 mm).

Synthesis of [K(222)][Co(TPP)(2-MeIm⁻)]: [Co(TPP)] (10 mg, 0.015 mmol) was dried in vacuum for 1 h. Excess [K(2-MeIm⁻)] (6 mg, 0.050 mmol) and Kryptofix 222 (17 mg, 0.045 mmol) in THF (5 mL) was added to the [Co(TPP)] solid by cannula. The mixture was stirred for 2 h and transferred into glass tubes (8 mm × 250 mm) which were layered with hexanes as nonsolvent. Several weeks, X-ray quality crystals were collected.

Synthesis of [K(222)][Co(TTP)(2-MeIm⁻)]: Similar reaction procedures to above were performed except using heptane as the nonsolvent.

X-ray Structure Determinations: Single crystal experiments were carried out on a BRUKER D8 QUEST system with graphite-monochromated Mo Kα radiation (λ = 0.71073 Å). The crystal samples were placed in inert oil, mounted on a glass fiber attached to a brass mounting pin, and transferred to the cold dinitrogen gas steam (100 K). Crystal data were collected and integrated using a Bruker Apex II system. The structures were solved by direct method (SHELXS-2014) and refined against F2 using SHELXL-2014.[12] Subsequent difference Fourier syntheses led to the location of all remaining nonhydrogen atoms. All nonhydrogen atoms were refined anisotropically, and hydrogen atoms were idealized with the standard SHELX idealization methods if not remarked upon otherwise below. For the structure refinement, all data were used including negative intensities. The program SADABS [13] was used to apply the absorption correction. Complete crystallographic details, atomic coordinates, anisotropic thermal parameters, and fixed hydrogen atom coordinates are given in Crystallographic information files (CIFs); a brief summary of crystallographic details is given in Table S1.

[Co(TPP)(2-MeHIm)]·0.9THF. A purple block-shaped crystal with dimensions of 0.15 × 0.21 × 0.40 mm^3 was used for the structure determination. The asymmetric unit contains a half porphyrin and a 0.45 tetrahydrofuran solvent molecule. There is a crystallographic 2-fold axis passing through the cobalt atom. The axial 2-methylimidazole ligand and the THF solvent molecule are disordered between two 2-fold related sites. Two outliers were omitted in the last cycles of refinement.

[Co(TTP)(2-MeHIm)]·PhCl·0.5C_6H_{14}. A purple block-shaped crystal with dimensions of 0.26 × 0.34 × 0.43 mm^3 was used for the structure determination. The asymmetric unit contains one porphyrin complex, one fully occupied chlorobenzene and one half hexanes molecule. The chlorobenzene molecule is disordered over two positions, and the site occupancy factors (SOFs) of disordered moieties are refined by means of a “free variable”. The final SOFs are 0.66 and 0.34. One carbon atom (C2S) on the chlorobenzene exhibited unusual thermal motions, thus this atom was restrained by “ISOR” command. Two outliers were omitted in the last cycles of refinement.

[K(222)][Co(TPP)(2-MeIm⁻)]·THF. A black block-shaped crystal with dimensions of 0.28 × 0.30 × 0.34 mm^3 was used for the structure determination. The asymmetric unit contains one porphyrin complex, one potassium cation chelated in Kryptofix 222 and one disordered THF solvent molecule. The final SOFs of the disordered THF molecule by using refinement of a “free variable” are 0.72 and 0.28. The five atoms (O1SA, C1SA, C2SA, C3SA, C4SA) of the THF were restrained by “similar U_{ij}” (SIMU) to constrain the anisotropic displacement parameters (ADP). Eight outliers were omitted in the last cycles of refinement.

[K(222)][Co(TTP)(2-MeIm⁻)]·THF. A black block-shaped crystal with dimensions of 0.03 × 0.19 × 0.24 mm^3 was used for the structure determination. The asymmetric unit contains one porphyrin complex, one potassium cation chelated in Kryptofix 222 and one THF solvent molecule. The imidazolate ligand is disordered over two positions and refined by dint of a “free variable”, and the final SOFs of disordered moieties are 0.66 and 0.34. The five atoms (N5A, N6A, C29A, C30A, C31A) of the imidazolate ring were restrained by “similar U_{ij}” (SIMU) to constrain ADPs. Five atoms (N6, C31, C29A, C31A, C32A) on the imidazolate and two atoms

(C3S, C4S) on the THF molecule exhibited unusual thermal motions, thus the seven atoms were restrained by “ISOR” commands. Four outliers were omitted in the last cycles of refinement.

Supplementary Table 1. Complete Crystallographic Details for [Co(TPP)(2-MeHIm)]·0.9THF, [Co(TTP)(2-MeHIm)]·PhCl·0.5C_6H_{14}, [K(222)][Co(TPP)(2-MeIm^{-})]·THF, [K(222)][Co(TTP)(2-MeIm^{-})]·THF.

	[Co(TPP)(2-MeHIm)]·0.9THF	[Co(TTP)(2-MeHIm)]·PhCl·0.5C_6H_{14}	[K(222)][Co(TPP)(2-MeIm^{-})]·THF	[K(222)][Co(TTP)(2-MeIm^{-})]·THF
Chemical formula	$C_{51.60}H_{41.20}CoN_6O_{0.90}$	$C_{61}H_{55}ClCoN_6$	$C_{70}H_{77}CoKN_8O_7$	$C_{74}H_{85}CoKN_8O_7$
FW	818.63	966.49	1240.42	1296.53
a, Å	17.1642(6)	13.9316(5)	21.3400(9)	11.2977(4)
b, Å	16.0230(6)	22.6359(7)	12.7274(5)	15.5368(6)
c, Å	14.6045(5)	15.3909(5)	25.1482(11)	19.9818(7)
α, deg	90	90	90	78.6764(12)
β, deg	98.5080(11)	97.1030(10)	112.8594(14)	75.6626(12)
γ, deg	90	90	90	81.0220(11)
V, Å^3	3972.4(2)	4816.3(3)	6293.9(5)	3310.6(2)
space group	*C2/c*	$P2_1/c$	$P2_1/c$	*P*–1
Z	4	4	4	4
temp, K	100(2)	100(2)	100(2)	100(2)
D_{calcd}, g cm^{-3}	1.369	1.333	1.309	1.301
μ, mm^{-1}	0.481	0.460	0.400	0.384
final *R* indices	R_1 = 0.0315	R_1 = 0.0431	R_1 = 0.0406	R_1 = 0.0516
[$I > 2\sigma(I)$]	wR_2 = 0.0781	wR_2 = 0.1018	wR_2 = 0.0968	wR_2 = 0.1060
final *R* indices	R_1 = 0.0395	R_1 = 0.0578	R_1 = 0.0570	R_1 = 0.0902
(all data)	wR_2 = 0.0829	wR_2 = 0.1100	wR_2 = 0.1079	wR_2 = 0.1204

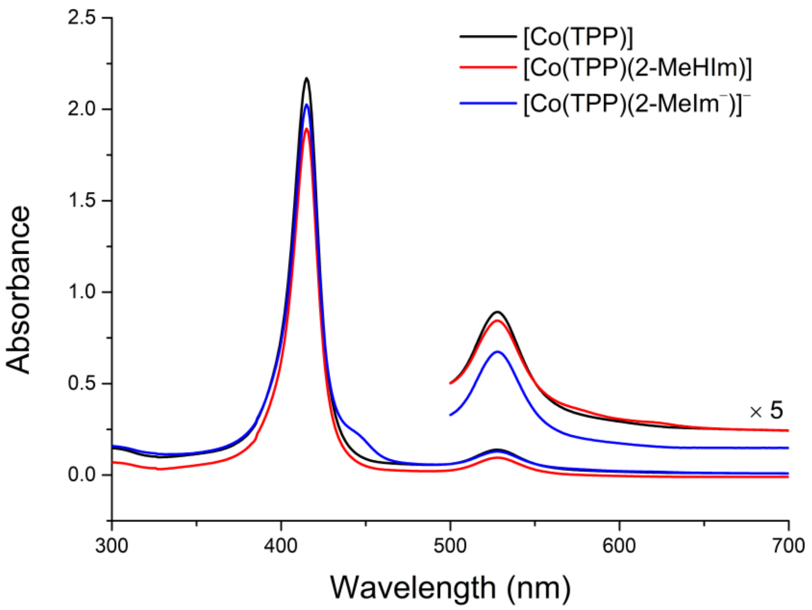

Supplementary Figure 1. UV-Vis spectrum of THF solution of [Co(TPP)] (black), [Co(TPP)(2-MeHIm)] (red) [Co(TPP)(2-MeIm⁻)]⁻ (blue). The spectra from 500 to 700 nm are enlarged by 5 times. (the UV-vis spectra of [Co(TPP)(2-MeIm⁻)]⁻ was measured by mixing 1 equivalent of [K(222)(2-MeIm⁻)] and [Co(TPP)]).

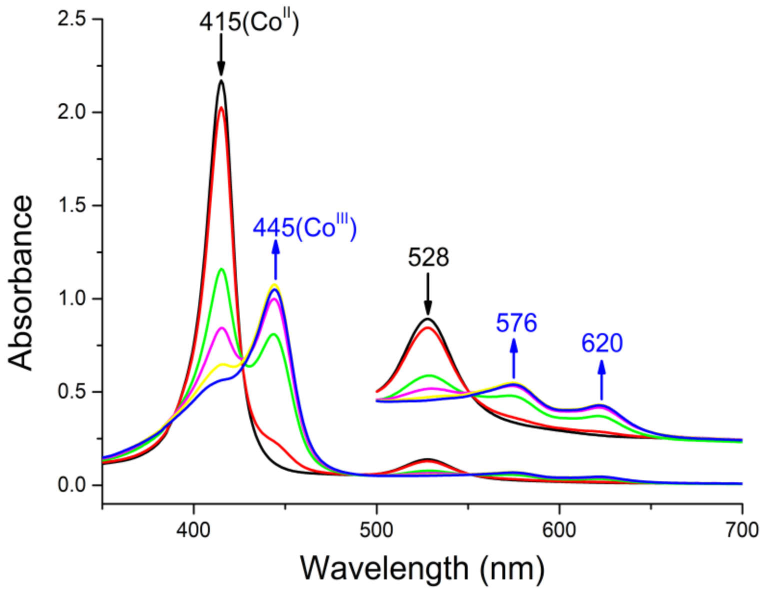

Supplementary Figure 2. UV-vis spectral change (in THF at 295 K) of 7.45 × 10^{-5} M solution of [Co^{II}(TPP)] upon addition of 0, 1, 3, 5, 10, 20 equiv. of [K(222)(2-MeIm⁻)].

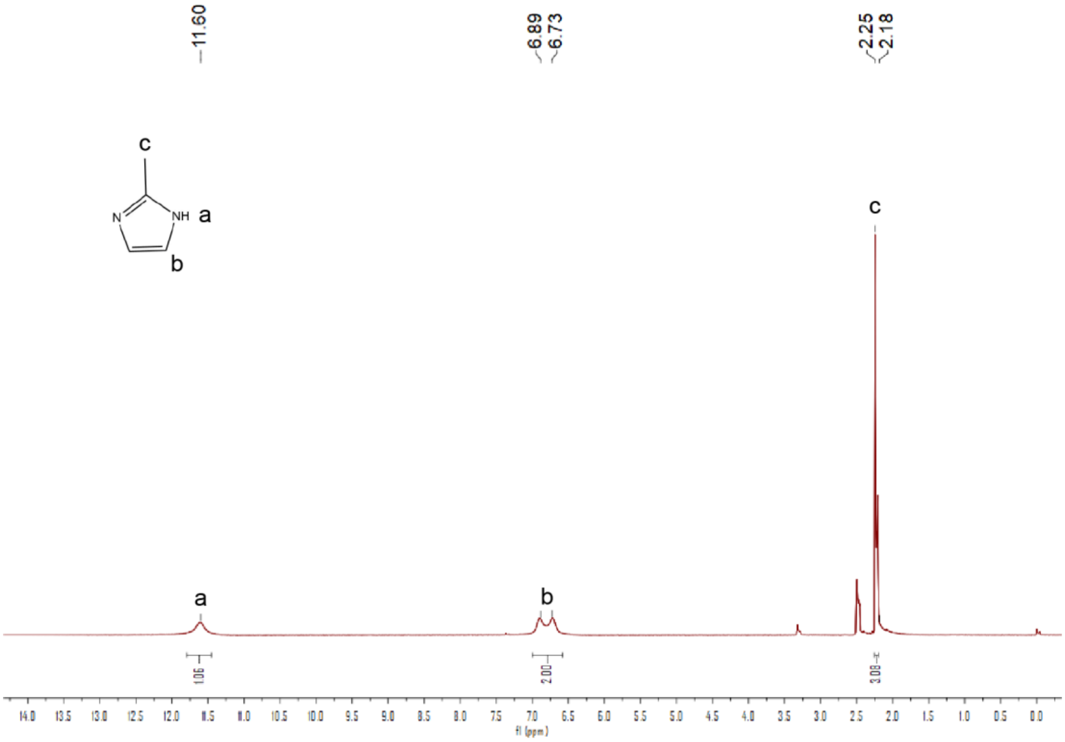

Supplementary Figure 3. ^{1}H NMR spectra of 2-MeHIm in DMSO-d6, 400 MHz.

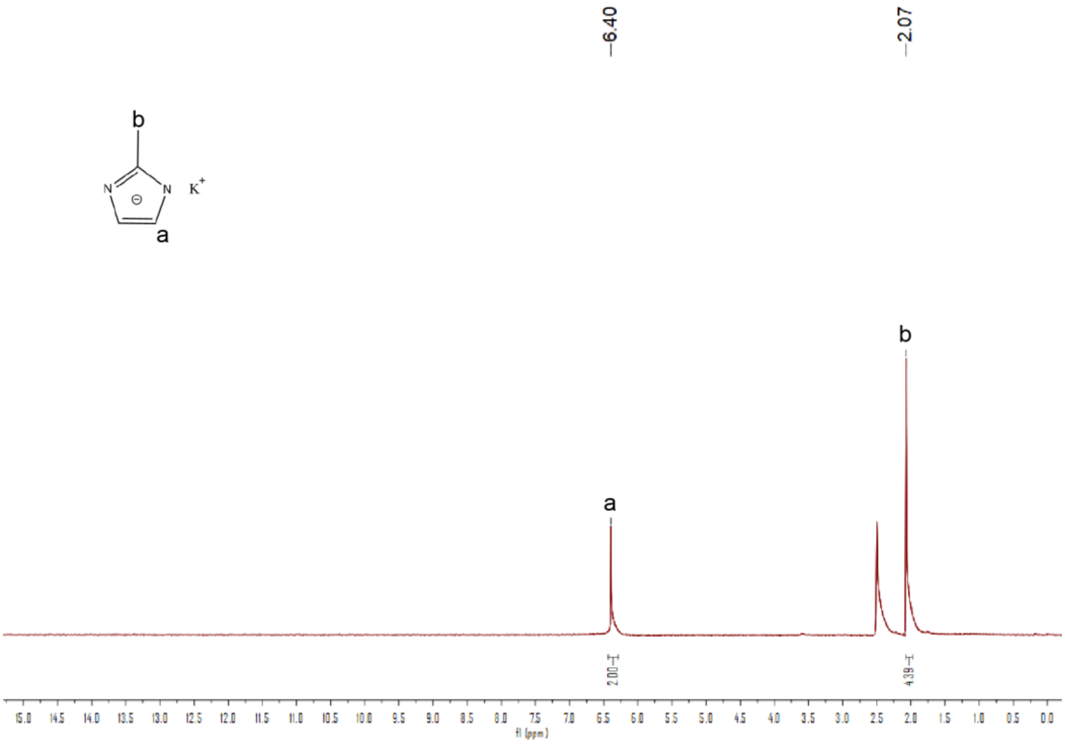

Supplementary Figure 4. ^{1}H NMR spectra of 2-MeImK in DMSO-d6, 400 MHz.

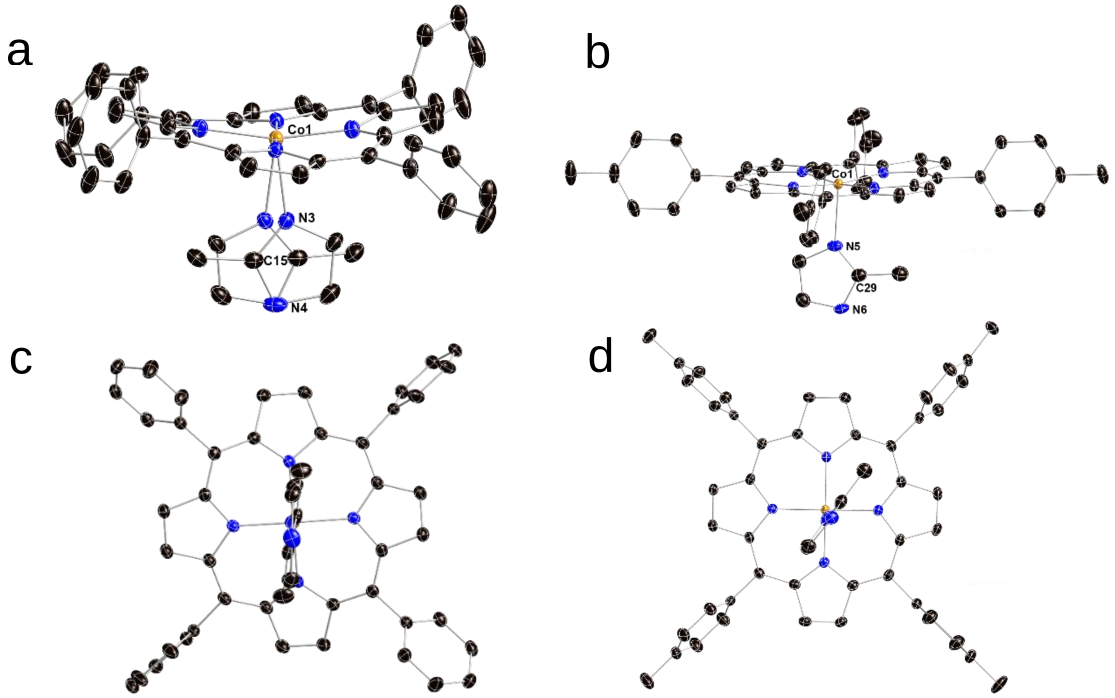

Supplementary Figure 5. Thermal ellipsoid diagrams of [Co(TPP)(2-MeHIm)] (a, c), [Co(TTP)(2-MeHIm)] (b, d). Thermal ellipsoids of all atoms are contoured at the 50% probability level. Hydrogen atoms and solvent molecules are not shown for clarity.

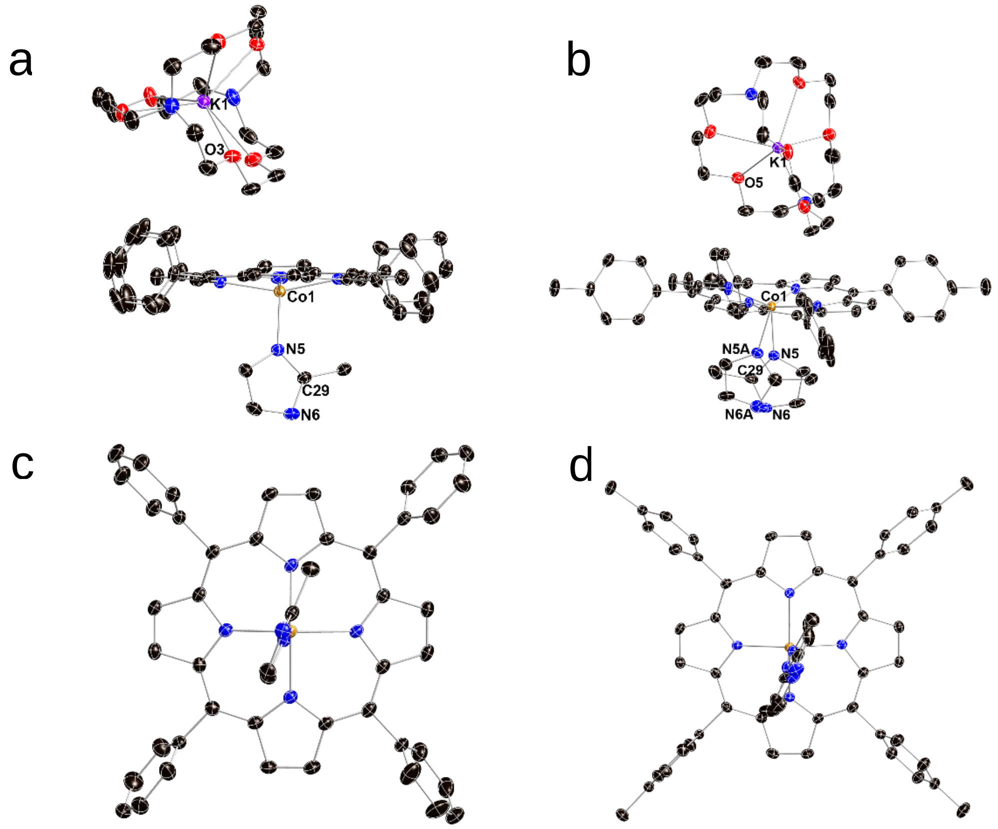

Supplementary Figure 6. Thermal ellipsoid diagrams of [Co(TPP)(2-MeIm^{-})]$^{-}$ (a, c) and [Co(TTP)(2-MeIm^{-})]$^{-}$ (b, d). Thermal ellipsoids of all atoms are contoured at the 50% probability level. Hydrogen atoms and solvent molecules are not shown for clarity.

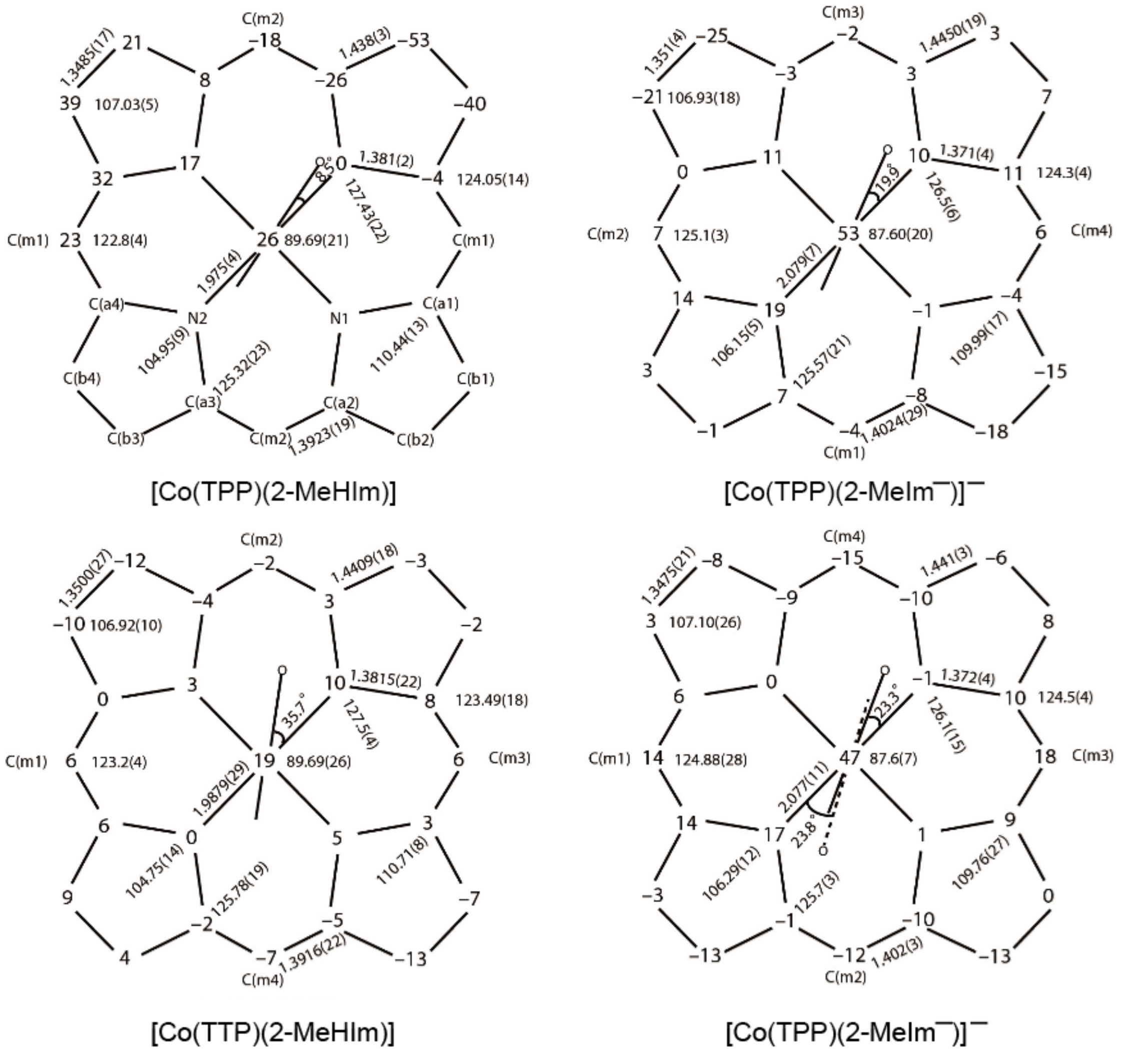

Supplementary Figure 7. Formal diagram of the porphyrin cores of [Co(TPP)(2-MeHIm)], [Co(TTP)(2-MeHIm)], [Co(TPP)(2-MeIm⁻)]⁻, [Co(TTP)(2-MeIm⁻)]⁻. Averaged values of the chemically unique bond distances (in Å) and angles (in degree) are shown. The numbers in parentheses are the esd's calculated on the assumption that the averaged values were all drawn from the same population. The perpendicular displacements (in units of 0.01 Å) of the porphyrin core atoms from the 24-atom mean plane are also displayed. The circle represents the position of the methyl groups on each ligand. Positive values of the displacements are towards the axial ligand.

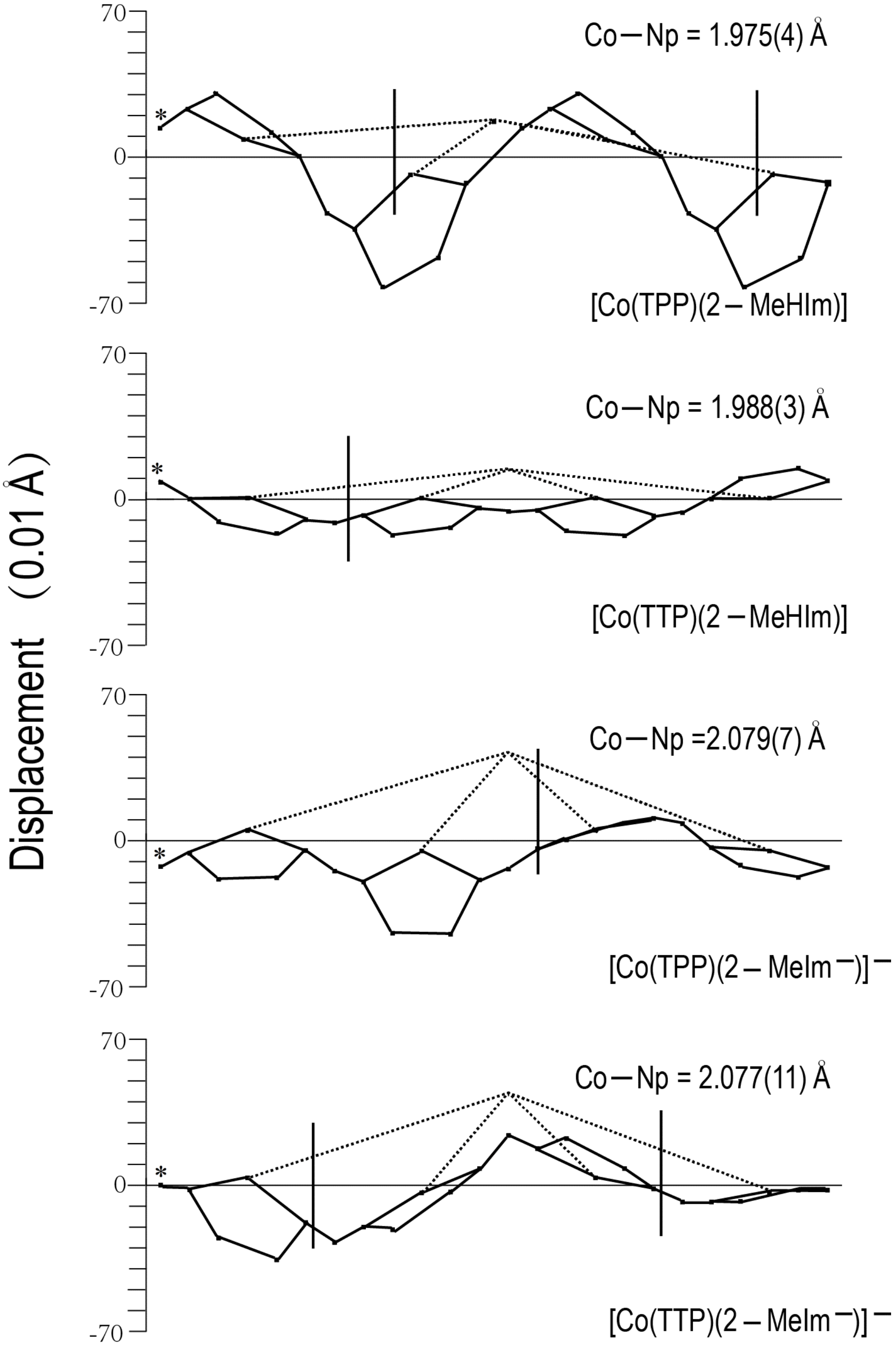

Supplementary Figure 8. “Shoestring” diagrams illustrating the core conformations and cobalt displacements of two imidazole–ligated cobalt(II) porphyrinates and two imidazolate–ligated cobalt(II) porphyrinates. The displacements from the cobalt atom and the atoms of the porphyrin core from the mean plane defined by the pyrrole four nitrogen atoms are given. The position of the axial imidazole or imidazolate ligand with respect to directions defined by the Co–Np directions is shown. All the diagrams begin with C(m1) (the asterisk) and finish at C(α8) atoms.

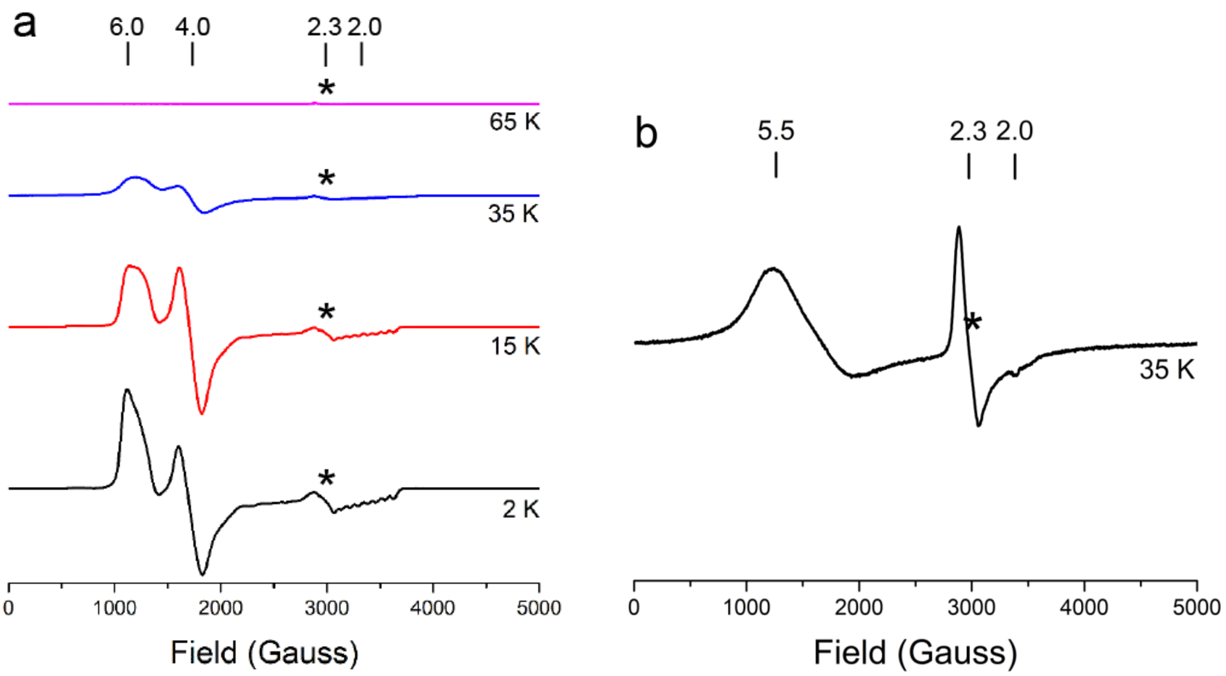

Supplementary Figure 9. Multi-temperature EPR spectra of crystalline $[Co(TPP)(2\text{-}MeIm^{-})]^{-}$. a: the sample was grinded inside the EPR tube by a quartz pestle. Signals at 6.0, .4.0, 2.0 belong to high-spin $[Co(TPP)(2\text{-}MeIm^{-})]^{-}$. The signal at 2.3 is suppsoed to be $[Co(TPP)\cdots(2\text{-}MeIm^{-})]^{-}$ intermediate which was generated during reaction and accompanied the isolated crystals through the mother liquor and/or was generated during grinding process. b: the sample was grinded by the agate mortar. The signal at 5.5 belongs to high-spin $[Co(TPP)(2\text{-}MeIm^{-})]^{-}$. The strong signal at 2.3 suggests vigorous grinding has produced more $[Co(TPP)\cdots(2\text{-}MeIm^{-})]^{-}$ intermediate. The signal at 2.0 is supposed to be a radical which is generated during the oxidation of Co(II) to Co(III) upon addition of imidazolate (Supplementary Figure 2). The mechanism studies are underway which will be reported in a separate paper.

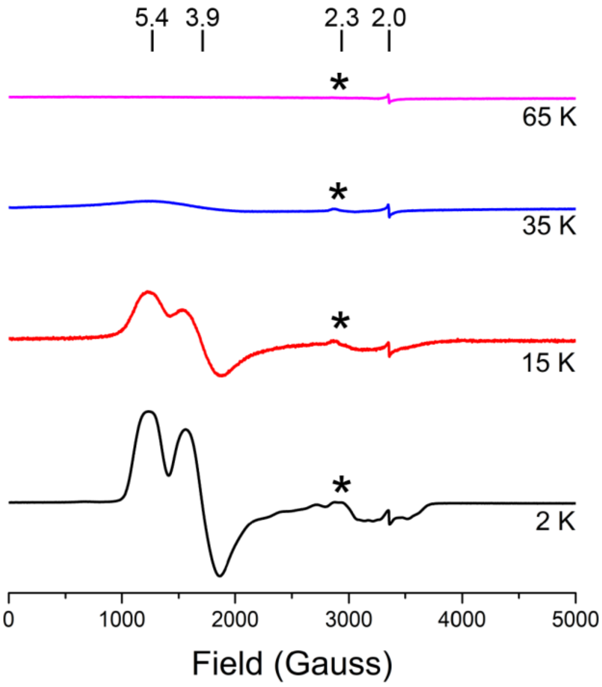

Supplementary Figure 10. Multi-temperature EPR spectra of $[Co(TTP)(2\text{-}MeIm^{-})]^{-}$ (crystalline) at 2, 15, 35 and 65 K. See footnote of Supplementary Figure 9 for explanations on the radical signal at 2.0.

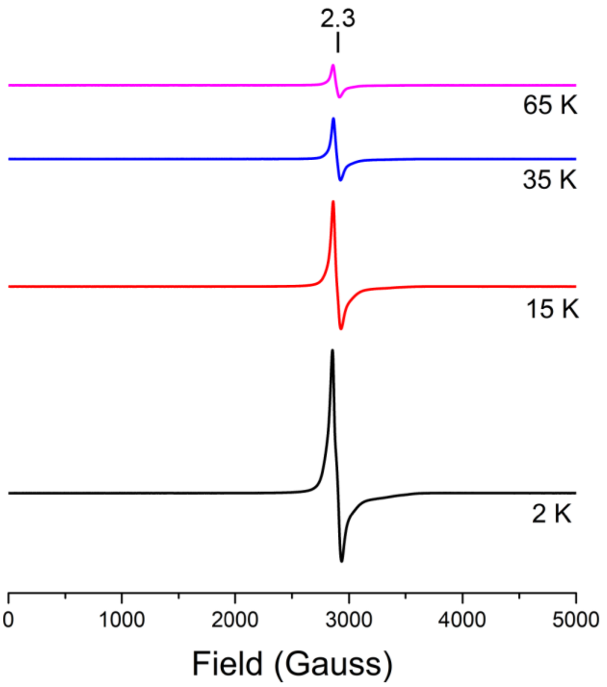

Supplementary Figure 11. Multi-temperature EPR spectra of [Co(TPP)(2-MeHIm)] (crystalline) at 2, 15, 35 and 65 K.

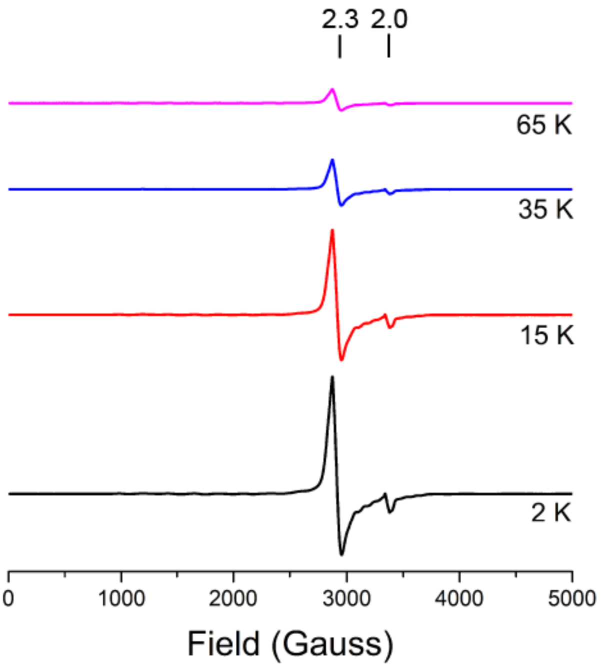

Supplementary Figure 12. Multi-temperature EPR spectra of [Co(TTP)(2-MeHIm)] (crystalline) at 2, 15, 35 and 65 K. The radical signal at g = 2.0 is supposed to be generated due to sample grinding process and/or decomposition.

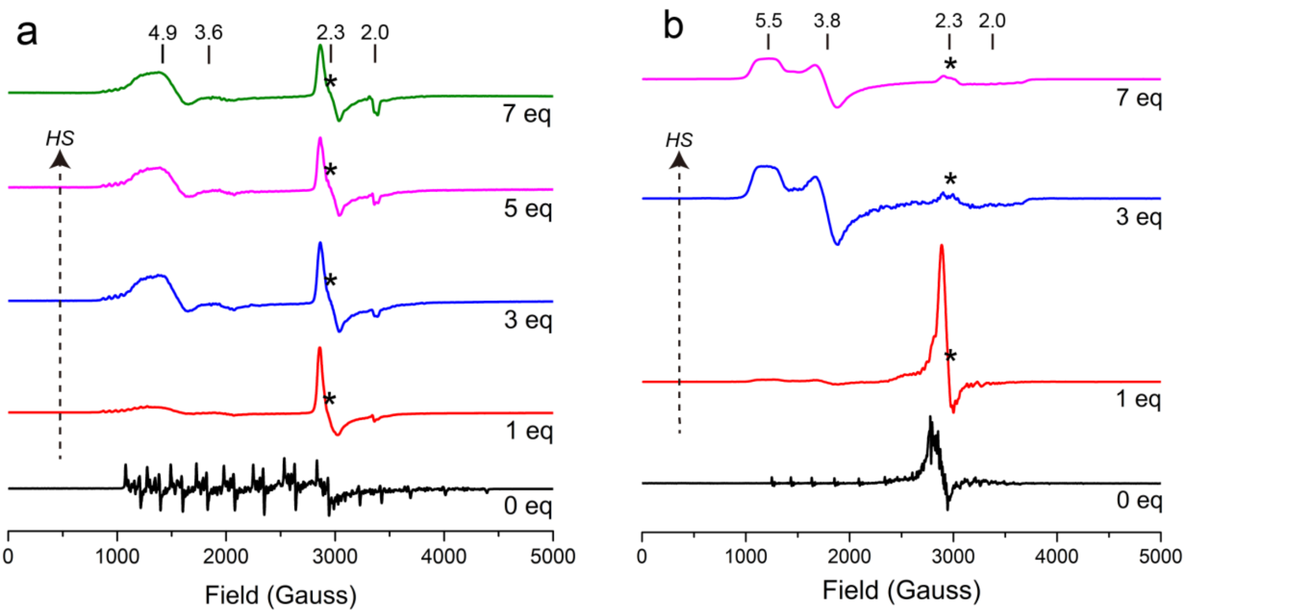

Supplementary Figure 13. EPR spectra of [Co(TPP)] with different equivalents of [K(222)(2-MeIm⁻)] in PhCl (a) and THF solution[14] (b) at 4 K. (c = 2.98 × 10^{-3} mmol/mL). See footnote of Supplementary Figure 9 for explanations on the radical signal at 2.0.

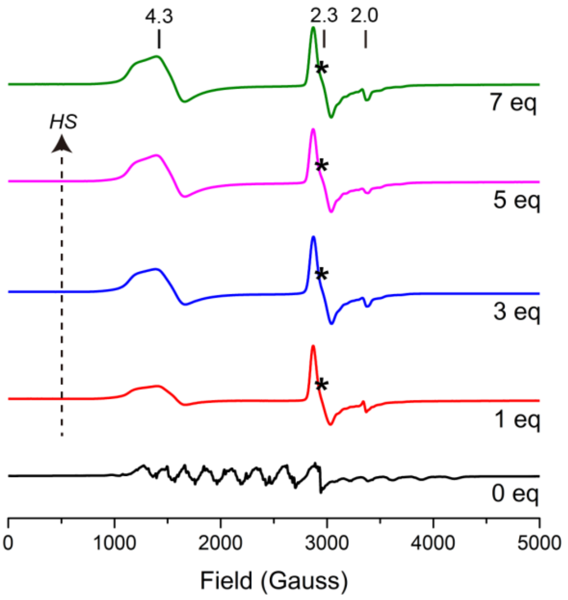

Supplementary Figure 14. EPR spectra of [Co(TTP)] with different equivalents of [K(222)(2-MeIm⁻)] in PhCl at 4 K (frozen solution, c = 2.98 × 10^{-3} mmol/mL). See footnote of Supplementary Figure 9 for explanations on the radical signal at 2.0.

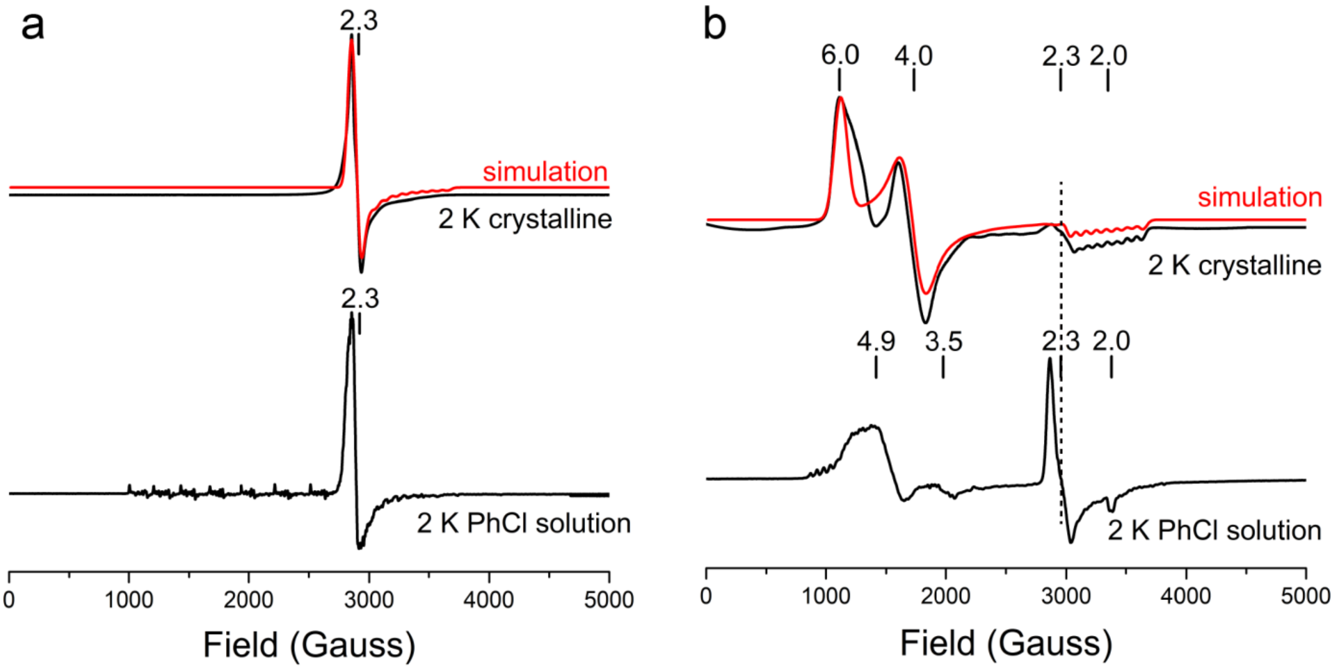

Supplementary Figure 15. Experimental (PhCl solution and crystalline) and simulated X-band EPR spectra of [Co(TPP)(2-MeHIm)] (a, frozen solution, c = 2.98 × 10^{-3} mmol/mL, CoTPP : 2-MeHIm = 1 : 10) and $[Co(TPP)(2\text{-MeIm}^{-})]^{-}$ (b, frozen solution, c = 2.98 × 10^{-3} mmol/mL, CoTPP : 2-MeIm^{-} = 1 : 3). See footnote of Supplementary Figure 9 for explanations on the radical signal at 2.0.

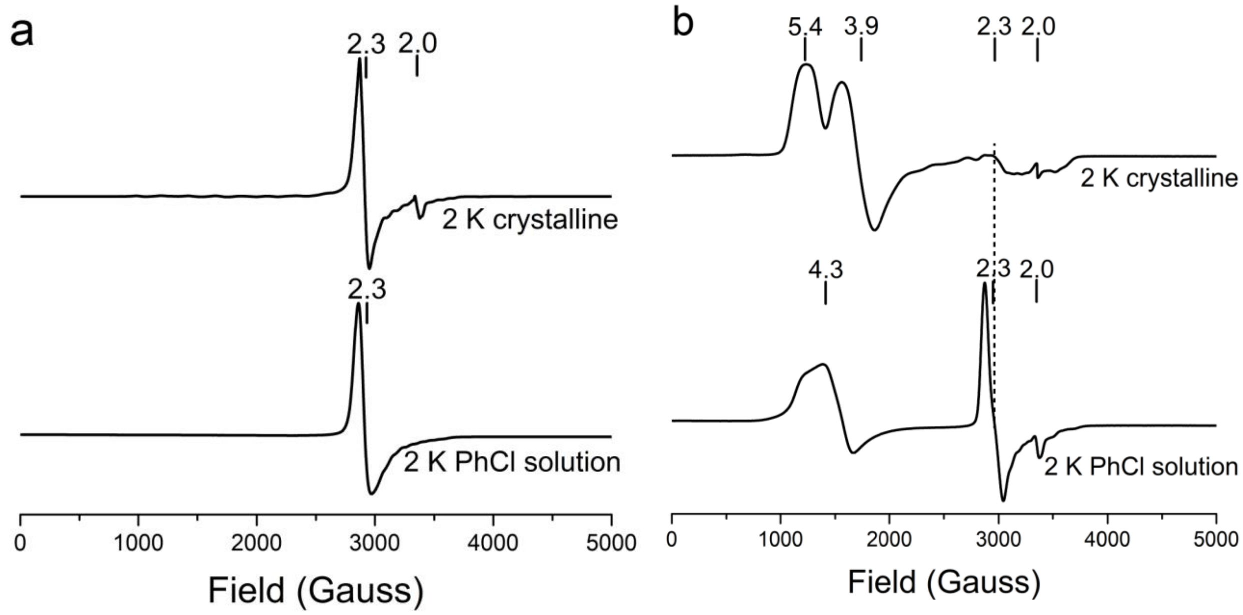

Supplementary Figure 16. Experimental (PhCl solution and crystalline) X-band EPR spectra of [Co(TTP)(2-MeHIm)] (a, frozen solution, c = 2.98 × 10^{-3} mmol/mL, CoTTP : 2-MeHIm = 1 : 10) and $[Co(TTP)(2\text{-MeIm}^{-})]^{-}$ (b, frozen solution, c = 2.98 × 10^{-3} mmol/mL, CoTTP : 2-MeIm^{-} = 1 : 3). See footnote of Supplementary Figure 9 for explanations on the radical signal at 2.0.

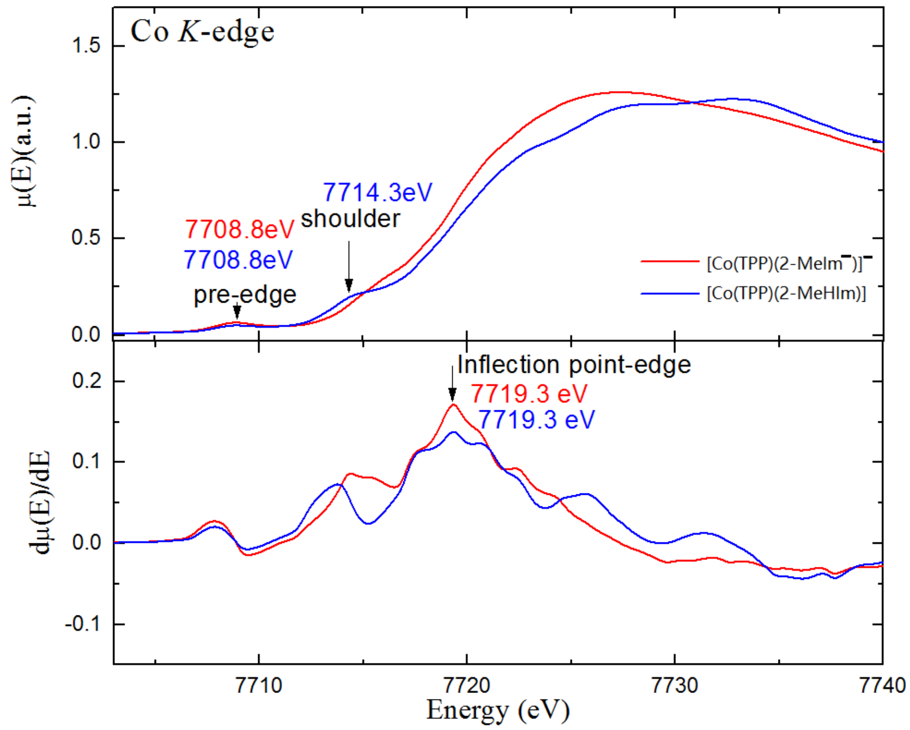

Supplementary Figure 17. Co *K*-edge XANES of [Co(TPP)(2-MeHIm)] and [Co(TPP)(2-MeIm$^-$)]$^-$.

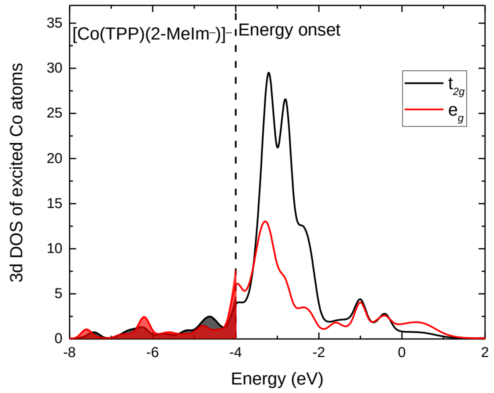

Supplementary Figure 18. Projected partial density of states for the Co 3d orbitals; the onset of the energy marking the unoccupied states are calibrated from the experimental spectra.

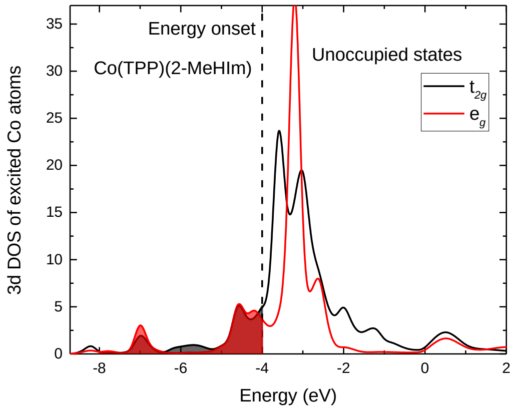

Supplementary Figure 19. Projected partial density of states for the Co 3d orbitals; the onset of the energy marking the unoccupied states are calibrated from the experimental spectra.

Supplementary Table 2. FWHM of the unoccupied states of t_{2g} and e_g

	t_{2g}	FWHM-t_{2g}	e_g	FWHM-e_g	t_{2g}/e_g
[Co(TPP)(2-MeIm⁻)]⁻	35.45	0.72	19.20	1.22	1.84
[Co(TPP)(2-MeHIm)]	30.19	0.84	21.63	0.23	1.39

As shown in Figure.S13 and Figure.S14, the unoccupied states projected on the t_{2g} and e_g orbitals are given. In Table S2, the unoccupied states were integrated from -4 to 2 eV, corresponding to the pre-edge region. The ratio of t_{2g} to e_g is 1.4 and 1.8 for [Co(TPP)(2-MeHIm)] and [Co(TPP)(2-MeIm⁻)]⁻ respectively. As for simple electronic configuration picture, the high spin (HS) configuration of Co d^7 left 2 e_g empty states and 1 t_{2g} empty states. By contrast, the low spin (LS) configuration gives 3 e_g empty states while no t_{2g} empty states. Qualitatively, the high spin configuration has more t_{2g} empty states than low spin configuration. Hence, it is safe to conclude that [Co(TPP)(2-MeIm⁻)]⁻ is in high spin while [Co(TPP)(2-MeHIm)] is in low spin states, given the more t_{2g} empty states in [Co(TPP)(2-MeIm⁻)]⁻.

Supplementary Table.3. Structural parameters of the Co-N first shell coordination, e.g. bond distance, coordination

	No. Atom	Co-N. bond	Δr [Å]	σ^{2} [Å^{2}]	Coordination	Energy	R-factor	Symmetry
[Co(TPP)(2-MeIm^{-})]$^{-}$	0.95N	1.8207	-0.2125	0.0056	5.7±0.5	5	0.0009	Lower
	0.95N	2.0232	-0.0444	0.0056				
	0.95N	2.0350	-0.0447	0.0056				
	1.43N	2.0387	-0.0448	0.0056				
	1.43N	2.0391	-0.0448	0.0056				
[Co(TPP)(2-MeHIm)]	1.9 N	1.9390	-0.0322	0.0051	4.94±0.5	5	0.0001	Higher
	1.9 N	1.9459	-0.0323	0.0051				
	1.14N	2.3638	0.1870	0.0114				

No. Atom XN refers that there are X nitrogen atoms in this path; e.g. 0.95N means 0.95 nitrogen atoms in this Co-N bond; Co-N bond distance is the bond distance extracted from EXAFS fitting; Δr is the difference of EXAFS fitting with respect to the single crystal results; Coordination number is the total number of coordinated atoms in the first shell surrounding the central absorber; Energy shift is the shift of absorption energy; MSRD is the bond Debye-Waller factor; R-factor is the fitting criteria.

The fitting data are consistent with the single crystal structures, e.g. similarly long Co–N_p bond and either longer or shorter axial bond (Co–N_{Im} vs. Co–N_p) for [Co(TPP)(2-MeHIm)] or [Co(TPP)(2-MeIm^{-})]$^{-}$ resepctively. The higher coordination symmetry of [Co(TPP)(2-MeIm^{-})]$^{-}$ (vs. [Co(TPP)(2-MeHIm)]) which gives more degenerate states is in agreement with its larger pre-edge intensity of experimental spectra.[15]

TDDFT_XANES

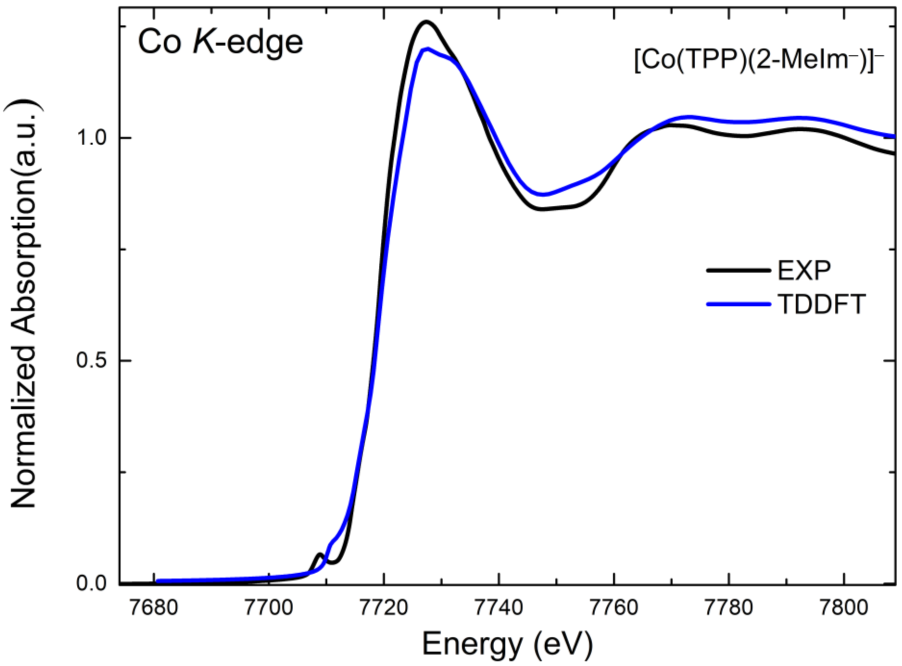

Supplementary Figure 20. Comparison of experimental (black) and TDDFT(blue) for [Co(TPP)(2-MeIm$^-$)]$^-$.

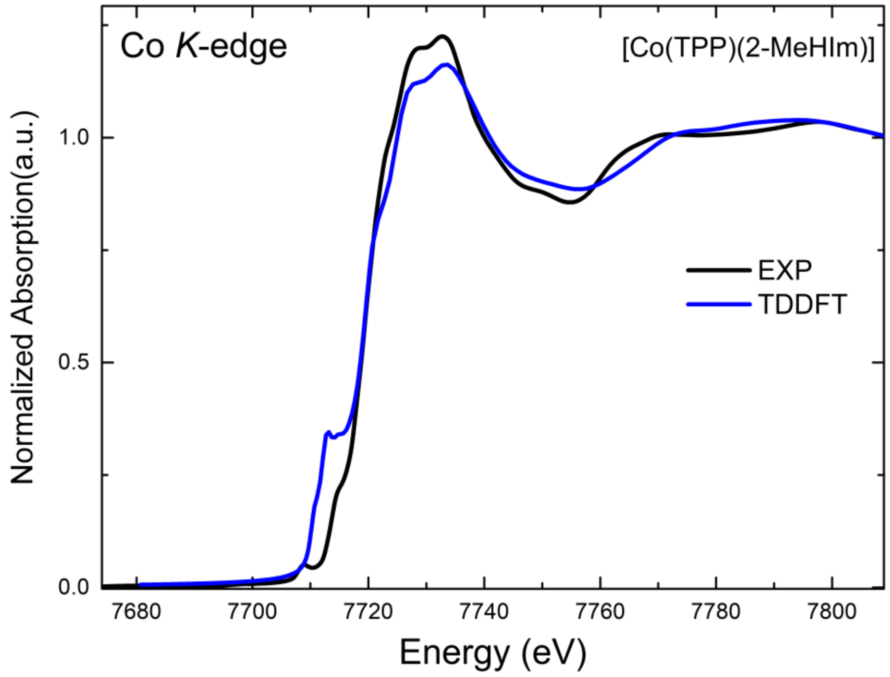

Supplementary Figure 21. Comparison of experimental (black) and TDDFT(blue) for [Co(TPP)(2-MeHIm)].

It is seen TDDFT underestimates the deep empty states when simulating the pre-edge feature of XANES spectra.

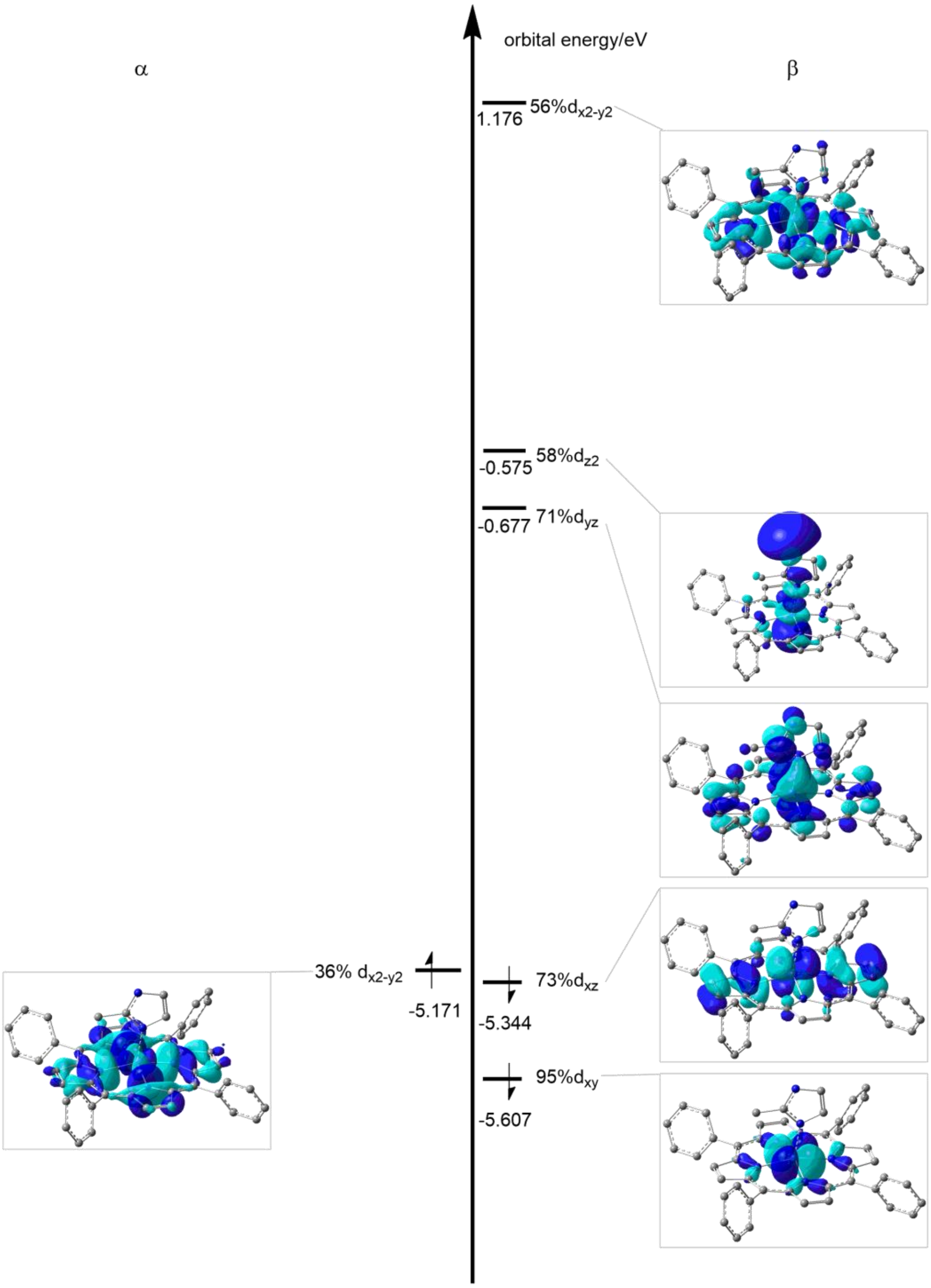

Supplementary Figure 22. Frontier Molecular Orbitals for [Co(TPP)(2-MeHIm)] with high spin S = 3/2 at UM06 level of theory. (α-orbitals shown in the left and β-orbitals shown in the right. Orbital pictures were visualized by Gaussview.)

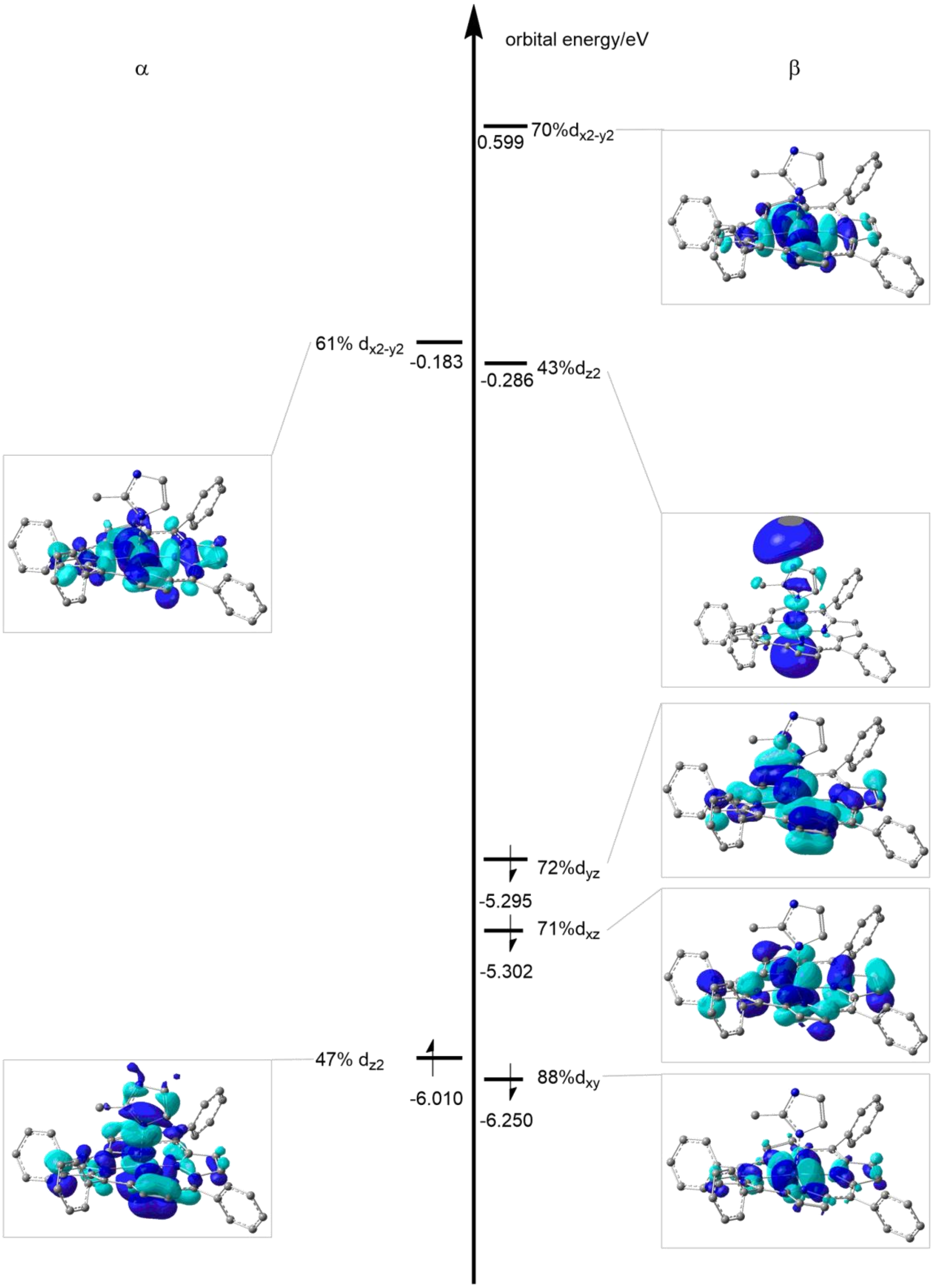

Supplementary Figure 23. Molecular Orbitals for [Co(TPP)(2-MeHIm)] with low spin S = 1/2 at UM06 level of theory.

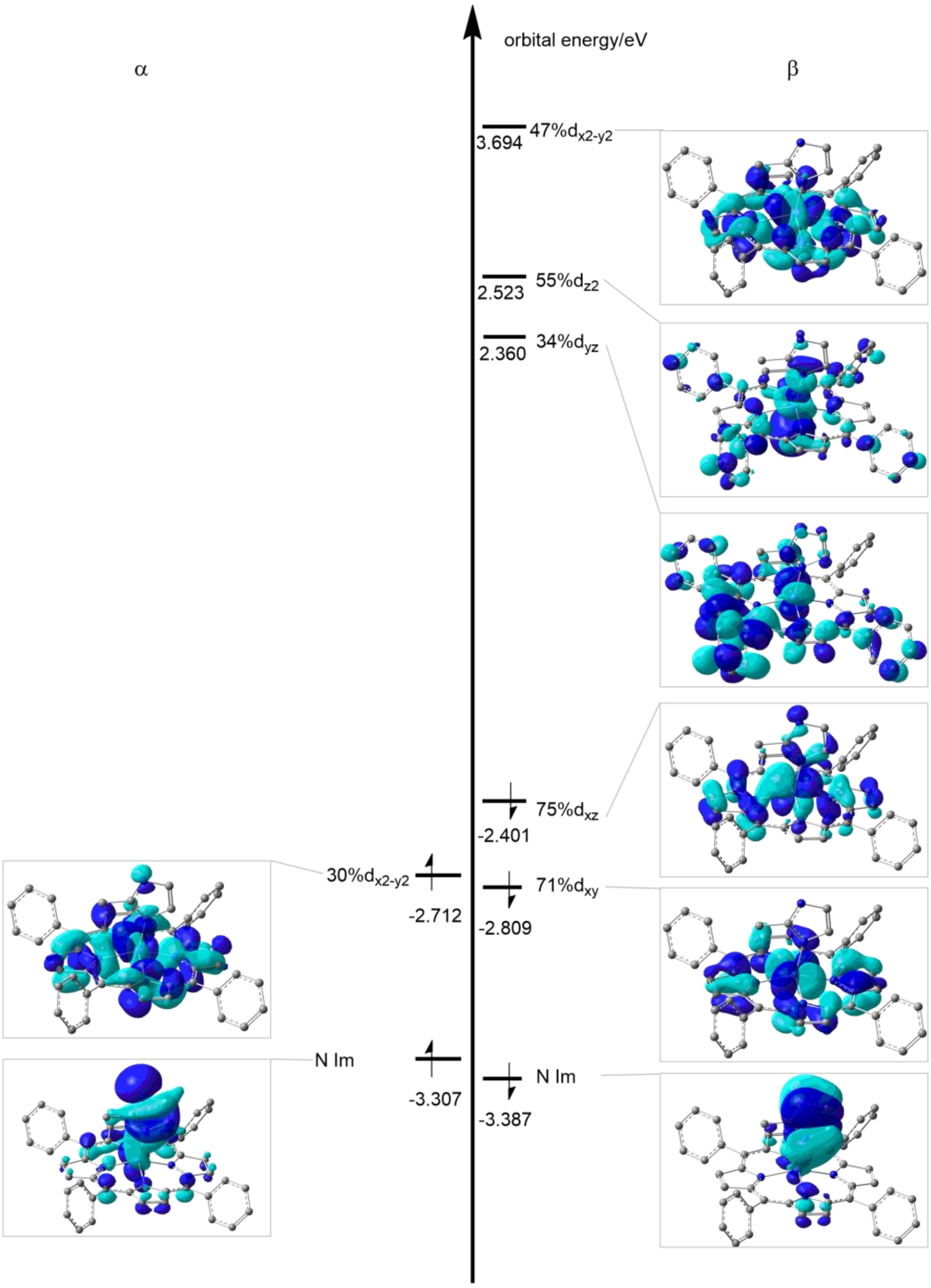

Supplementary Figure 24. Molecular Orbitals of [Co(TPP)(2-MeIm⁻)]⁻ with high spin S = 3/2 at UM06 level of theory.

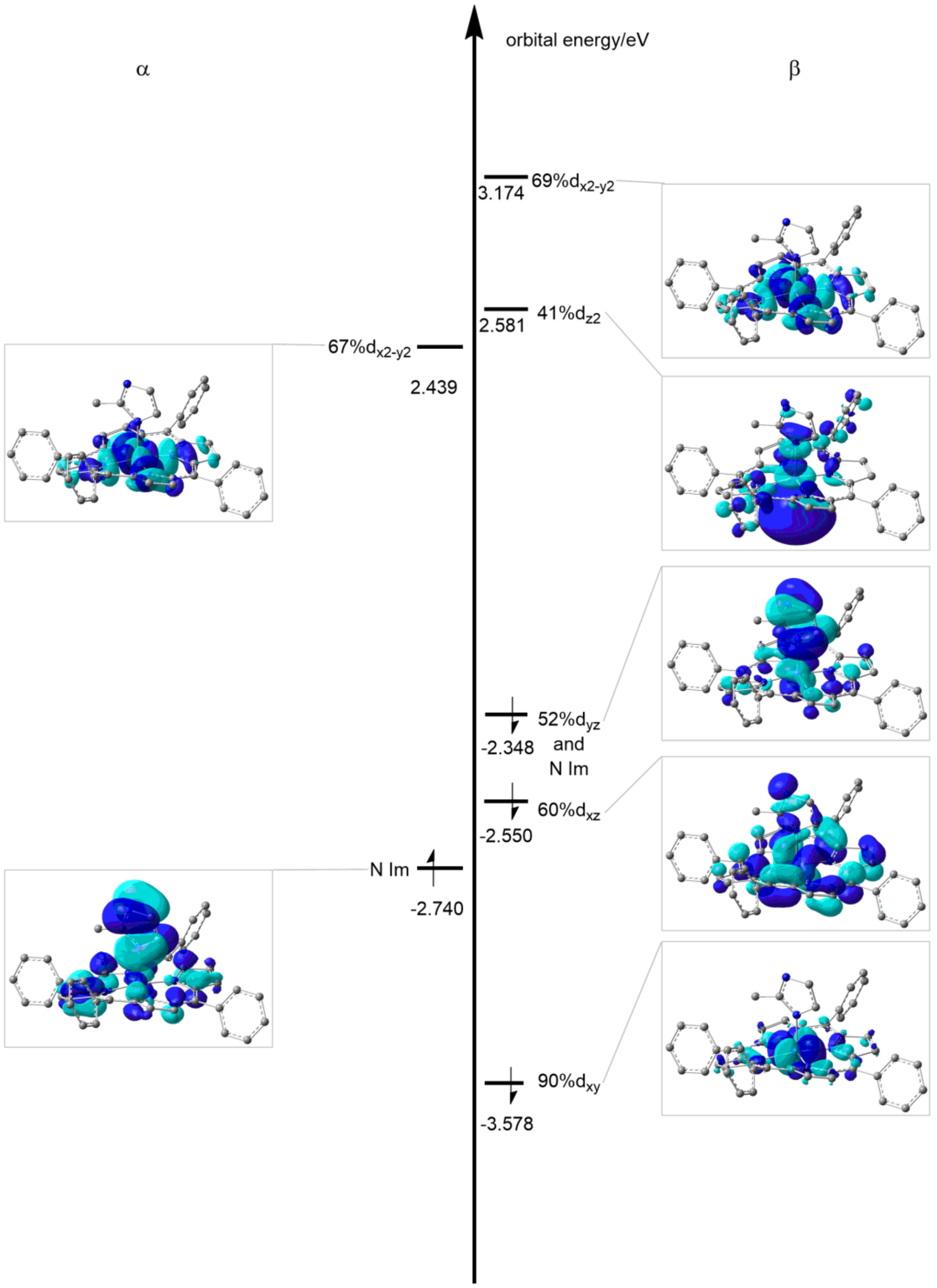

Supplementary Figure 25. Molecular Orbitals of [Co(TPP)(2-MeIm⁻)]⁻ with low spin S = 1/2 at UM06 level of theory.

Supplementary References

1 Hu, C. *et al.* Just a Proton: Distinguishing the Two Electronic States of Five-Coordinate High-Spin Iron(II) Porphyrinates with Imidazole/ate Coordination. *J. Am. Chem. Soc.* **132**, 3737-3750 (2010).

2 Adler, A. D. *et al.* A simplified synthesis for meso-tetraphenylporphine. *J. Org. Chem.* **32**, 476-476 (1967).

3 Adler, A. D., Longo, F. R., Kampas, F. & Kim, J. On the preparation of metalloporphyrins. *J. Inorg. Nucl. Chem.* **32**, 2443-2445 (1970).

4 Stoll, S. & Schweiger, A. EasySpin, a comprehensive software package for spectral simulation and analysis in EPR. *J. Magn. Reson.* **178**, 42-55 (2006).

5 Ravel, B. & Newville, M. ATHENA, ARTEMIS, HEPHAESTUS: data analysis for X-ray absorption spectroscopy using IFEFFIT. *J. Synchrotron. Radiat.* **12**, 537-541 (2005).

6 Rehr, J. J., Kas, J. J., Vila, F. D., Prange, M. P. & Jorissen, K. Parameter-free calculations of X-ray spectra with FEFF9. *Phys. Chem. Chem. Phys.* **12**, 5503-5513 (2010).

7 Frisch, M. J. *et al.* Gaussian 09, Revision D.01, Gaussian, Inc., Wallingford CT, 2009.

8 Zhao, Y. & Truhlar, D. G. The M06 suite of density functionals for main group thermochemistry, thermochemical kinetics, noncovalent interactions, excited states, and transition elements: two new functionals and systematic testing of four M06-class functionals and 12 other functionals. *Theor. Chem. Acc.* **120**, 215-241 (2008).

9 Becke, A. D. Density-functional thermochemistry. III. The role of exact exchange. *J. Chem. Phys.* **98**, 5648-5652 (1993).

10 Lee, C., Yang, W. & Parr, R. G. Development of the Colle-Salvetti correlation-energy formula into a functional of the electron density. *Phys. Rev. B* **37**, 785-789 (1988).

11 Weigend, F. & Ahlrichs, R. Balanced basis sets of split valence, triple zeta valence and quadruple zeta valence quality for H to Rn: Design and assessment of accuracy. *Phys. Chem. Chem. Phys.* **7**, 3297-3305 (2005).

12 Sheldrick, G. M.. A short history of SHELX. *Acta. Crystallogr. A* **64 (1)**, 112-122 (2008).

13 Krause, L., Herbst-Irmer, R., Sheldrick, G. M. & Stalke, D. Comparison of silver and molybdenum microfocus X-ray sources for single-crystal structure determination. *J. Appl. Cryst.* **48**, 3-10 (2015).

14 Baumgarten, M., Winscom, C. J. & Lubitz, W. Probing the surrounding of a cobalt(II) porphyrin and its superoxo complex by EPR techniques. *Appl. Magn. Reson.* **20**, 35-70 (2001).

15 Takashi, Y. Assignment of pre-edge peaks in K-edge x-ray absorption spectra of 3d transition metal compounds: electric dipole or quadrupole? *X-Ray Spectrom.* **37**, 572-584 (2008).
