# Supplementary material for: Proton mediated spin state transition of cobalt heme analogs
Source: Nat Commun. 2019 May 24;10:2303. doi: 10.1038/s41467-019-10357-z (PMC6534676; doi:10.1038/s41467-019-10357-z)
Supplement: Supplementary file 2 — Supplementary Data 1 [file 41467_2019_10357_MOESM2_ESM.docx]

**Supplementary data 1.** Calculated Cartesian Coordinates and Electronic Energy of [Co(TPP)(2-MeHIm)] and [Co(TPP)(2-MeIm^‒^)]^‒^ at UM06 and UB3LYP level of theory.

| [Co(TPP)(2-MeHIm)] S = 3/2 | |
| --- | --- |
| \| B3LYP \| \| E=-3561.123891 Hartree \| \| \| --- \| --- \| --- \| --- \| \| Co \| 0.0580104 \| 0.0672475 \| -0.1881950 \| \| N \| -0.9807466 \| 1.8413745 \| -0.4029520 \| \| N \| -1.7247566 \| -0.9516255 \| -0.5082790 \| \| C \| 0.9533934 \| 3.3874805 \| -0.2735560 \| \| C \| -3.2887726 \| 0.9675545 \| -0.6098030 \| \| C \| -0.4245406 \| 3.0965925 \| -0.3423100 \| \| C \| -2.3417456 \| 2.0067575 \| -0.5151890 \| \| C \| -2.9842566 \| -0.4094065 \| -0.5961700 \| \| C \| -1.8742726 \| -2.3166325 \| -0.4608470 \| \| C \| -1.4775116 \| 4.0917285 \| -0.3922350 \| \| H \| -1.3298886 \| 5.1612725 \| -0.3528170 \| \| C \| -2.6563316 \| 3.4209235 \| -0.5195940 \| \| H \| -3.6472956 \| 3.8409735 \| -0.6135980 \| \| C \| -3.9686076 \| -1.4721035 \| -0.5907820 \| \| H \| -5.0389426 \| -1.3342985 \| -0.6444030 \| \| C \| -3.2840896 \| -2.6484355 \| -0.5088300 \| \| H \| -3.6915916 \| -3.6491575 \| -0.4978220 \| \| C \| 1.3505414 \| 4.8272035 \| -0.1688660 \| \| C \| 1.1672444 \| 5.7071975 \| -1.2464500 \| \| H \| 0.7284264 \| 5.3294515 \| -2.1656600 \| \| C \| 1.5447014 \| 7.0470705 \| -1.1450190 \| \| H \| 1.3982524 \| 7.7137015 \| -1.9909340 \| \| C \| 2.1154104 \| 7.5279685 \| 0.0357690 \| \| H \| 2.4108004 \| 8.5708705 \| 0.1146160 \| \| C \| 2.3063724 \| 6.6604745 \| 1.1137130 \| \| H \| 2.7487574 \| 7.0266565 \| 2.0366260 \| \| C \| 1.9264684 \| 5.3212855 \| 1.0114730 \| \| H \| 2.0735604 \| 4.6442535 \| 1.8485140 \| \| C \| -4.7311906 \| 1.3504175 \| -0.7057550 \| \| C \| -5.4690036 \| 1.0768655 \| -1.8681910 \| \| H \| -4.9738156 \| 0.5879375 \| -2.7023700 \| \| C \| -6.8168326 \| 1.4275965 \| -1.9572410 \| \| H \| -7.3706296 \| 1.2113195 \| -2.8671170 \| \| C \| -7.4505506 \| 2.0595955 \| -0.8847710 \| \| H \| -8.4998046 \| 2.3338865 \| -0.9543720 \| \| C \| -6.7274526 \| 2.3373585 \| 0.2775450 \| \| H \| -7.2131296 \| 2.8256185 \| 1.1185430 \| \| C \| -5.3802036 \| 1.9843445 \| 0.3654620 \| \| H \| -4.8173276 \| 2.1984215 \| 1.2699600 \| \| N \| -0.1986256 \| -0.6418885 \| 4.0497230 \| \| N \| 1.0992714 \| -1.7166275 \| -0.5317760 \| \| N \| 1.8481264 \| 1.0795165 \| -0.4264350 \| \| C \| -0.8304856 \| -3.2646485 \| -0.4287410 \| \| C \| 3.4102714 \| -0.8340445 \| -0.6107540 \| \| C \| 0.5468244 \| -2.9720695 \| -0.4963140 \| \| C \| 2.4603934 \| -1.8764515 \| -0.6014860 \| \| C \| 3.1083124 \| 0.5402825 \| -0.5270720 \| \| C \| 1.9970064 \| 2.4414645 \| -0.3374230 \| \| C \| 1.6028024 \| -3.9663125 \| -0.5450300 \| \| H \| 1.4571284 \| -5.0367435 \| -0.5270020 \| \| C \| 2.7817714 \| -3.2904755 \| -0.6309680 \| \| H \| 3.7767804 \| -3.7051355 \| -0.7039170 \| \| C \| 4.0926424 \| 1.6036605 \| -0.4896150 \| \| H \| 5.1629704 \| 1.4673295 \| -0.5464630 \| \| C \| 3.4076414 \| 2.7755275 \| -0.3775100 \| \| H \| 3.8138284 \| 3.7758745 \| -0.3349710 \| \| C \| -1.2272886 \| -4.7058585 \| -0.3533970 \| \| C \| -1.0342066 \| -5.5666265 \| -1.4448220 \| \| H \| -0.5887646 \| -5.1719925 \| -2.3536220 \| \| C \| -1.4101586 \| -6.9086615 \| -1.3701150 \| \| H \| -1.2559646 \| -7.5597855 \| -2.2266340 \| \| C \| -1.9889706 \| -7.4117785 \| -0.2026290 \| \| H \| -2.2828696 \| -8.4564415 \| -0.1446240 \| \| C \| -2.1895036 \| -6.5640105 \| 0.8891870 \| \| H \| -2.6376156 \| -6.9476255 \| 1.8022430 \| \| C \| -1.8111566 \| -5.2227835 \| 0.8132850 \| \| H \| -1.9650826 \| -4.5612785 \| 1.6615450 \| \| C \| 4.8549244 \| -1.2148405 \| -0.6973400 \| \| C \| 5.6057024 \| -0.9145905 \| -1.8444010 \| \| H \| 5.1206134 \| -0.4050495 \| -2.6721950 \| \| C \| 6.9543424 \| -1.2644625 \| -1.9261950 \| \| H \| 7.5190094 \| -1.0267235 \| -2.8239580 \| \| C \| 7.5748894 \| -1.9224065 \| -0.8617290 \| \| H \| 8.6247454 \| -2.1958475 \| -0.9254910 \| \| C \| 6.8381774 \| -2.2270485 \| 0.2851770 \| \| H \| 7.3137014 \| -2.7356965 \| 1.1198550 \| \| C \| 5.4902224 \| -1.8746565 \| 0.3659690 \| \| H \| 4.9162464 \| -2.1103365 \| 1.2580860 \| \| N \| 0.1896444 \| -0.2166895 \| 1.9338220 \| \| C \| 1.3865444 \| -0.5402835 \| 2.5449030 \| \| H \| 2.3038514 \| -0.5559785 \| 1.9770250 \| \| C \| 1.1670444 \| -0.8082705 \| 3.8658600 \| \| H \| 1.8251194 \| -1.0867985 \| 4.6741090 \| \| H \| -0.7015466 \| -0.7609175 \| 4.9177870 \| \| C \| -0.7576676 \| -0.2851005 \| 2.8582980 \| \| C \| -2.2162606 \| -0.0523965 \| 2.6544530 \| \| H \| -2.7263176 \| 0.1126445 \| 3.6094310 \| \| H \| -2.3665676 \| 0.8198955 \| 2.0144720 \| \| H \| -2.6801446 \| -0.9066005 \| 2.1493040 \| | \| M06 \| \| E= -3559.295459 Hartree \| \| \| --- \| --- \| --- \| --- \| \| Co \| 0.0497432 \| -0.0007581 \| -0.2117285 \| \| N \| -0.3195538 \| 1.9959759 \| -0.5230995 \| \| N \| -1.9788148 \| -0.3633501 \| -0.5073475 \| \| C \| 2.0126312 \| 2.8215589 \| -0.4744975 \| \| C \| -2.7913548 \| 1.9584689 \| -0.5576325 \| \| C \| 0.6185022 \| 2.9941879 \| -0.4948375 \| \| C \| -1.5443158 \| 2.6073989 \| -0.5276815 \| \| C \| -2.9764828 \| 0.5670579 \| -0.5729725 \| \| C \| -2.5839798 \| -1.5877101 \| -0.5124995 \| \| C \| -0.0374658 \| 4.2762739 \| -0.4812195 \| \| H \| 0.4615122 \| 5.2381009 \| -0.4519675 \| \| C \| -1.3748388 \| 4.0373809 \| -0.5164695 \| \| H \| -2.1777298 \| 4.7655799 \| -0.5367625 \| \| C \| -4.2612748 \| -0.0914171 \| -0.6052245 \| \| H \| -5.2241488 \| 0.4042629 \| -0.6557155 \| \| C \| -4.0176238 \| -1.4270221 \| -0.5763515 \| \| H \| -4.7405288 \| -2.2344431 \| -0.6117145 \| \| C \| 2.8576292 \| 4.0461319 \| -0.4247275 \| \| C \| 2.9387972 \| 4.9092889 \| -1.5216535 \| \| H \| 2.3692962 \| 4.6735979 \| -2.4208605 \| \| C \| 3.7357692 \| 6.0480939 \| -1.4717015 \| \| H \| 3.7900572 \| 6.7073899 \| -2.3367845 \| \| C \| 4.4656752 \| 6.3408659 \| -0.3233805 \| \| H \| 5.0904282 \| 7.2319009 \| -0.2850235 \| \| C \| 4.3940052 \| 5.4883429 \| 0.7743325 \| \| H \| 4.9599172 \| 5.7126289 \| 1.6774895 \| \| C \| 3.5959162 \| 4.3505649 \| 0.7229525 \| \| H \| 3.5340302 \| 3.6811559 \| 1.5815435 \| \| C \| -4.0097498 \| 2.8136079 \| -0.5588405 \| \| C \| -4.8297618 \| 2.8942619 \| -1.6885085 \| \| H \| -4.5596738 \| 2.3243439 \| -2.5777035 \| \| C \| -5.9710448 \| 3.6892259 \| -1.6819965 \| \| H \| -6.5969898 \| 3.7420579 \| -2.5716105 \| \| C \| -6.3091348 \| 4.4188179 \| -0.5460995 \| \| H \| -7.2018298 \| 5.0423579 \| -0.5421075 \| \| C \| -5.5001118 \| 4.3483399 \| 0.5840855 \| \| H \| -5.7590648 \| 4.9148539 \| 1.4775235 \| \| C \| -4.3611018 \| 3.5504119 \| 0.5766015 \| \| H \| -3.7241028 \| 3.4929439 \| 1.4604215 \| \| N \| -0.0602318 \| 0.0049529 \| 4.0661475 \| \| N \| 0.4074402 \| -1.9988181 \| -0.5266185 \| \| N \| 2.0763972 \| 0.3629409 \| -0.5246465 \| \| C \| -1.9215978 \| -2.8250121 \| -0.4726275 \| \| C \| 2.8824522 \| -1.9592871 \| -0.5525225 \| \| C \| -0.5268318 \| -2.9985821 \| -0.4930485 \| \| C \| 1.6350532 \| -2.6071251 \| -0.5285565 \| \| C \| 3.0725922 \| -0.5682001 \| -0.5700175 \| \| C \| 2.6793342 \| 1.5860089 \| -0.5127965 \| \| C \| 0.1315302 \| -4.2795221 \| -0.4763975 \| \| H \| -0.3646218 \| -5.2426911 \| -0.4432415 \| \| C \| 1.4681572 \| -4.0373411 \| -0.5135245 \| \| H \| 2.2725542 \| -4.7638131 \| -0.5313355 \| \| C \| 4.3588442 \| 0.0905299 \| -0.5805055 \| \| H \| 5.3225922 \| -0.4050281 \| -0.6112545 \| \| C \| 4.1143312 \| 1.4263079 \| -0.5539885 \| \| H \| 4.8375882 \| 2.2339539 \| -0.5713485 \| \| C \| -2.7693398 \| -4.0472731 \| -0.4262955 \| \| C \| -2.8374978 \| -4.9189551 \| -1.5175575 \| \| H \| -2.2559488 \| -4.6907331 \| -2.4108785 \| \| C \| -3.6381358 \| -6.0552251 \| -1.4703025 \| \| H \| -3.6823658 \| -6.7204601 \| -2.3313885 \| \| C \| -4.3847978 \| -6.3380101 \| -0.3302365 \| \| H \| -5.0119288 \| -7.2274261 \| -0.2940035 \| \| C \| -4.3270528 \| -5.4771841 \| 0.7617585 \| \| H \| -4.9062178 \| -5.6932591 \| 1.6584635 \| \| C \| -3.5257708 \| -4.3417171 \| 0.7122545 \| \| H \| -3.4762208 \| -3.6655041 \| 1.5667575 \| \| C \| 4.0997162 \| -2.8159261 \| -0.5473955 \| \| C \| 4.9276292 \| -2.8915601 \| -1.6716245 \| \| H \| 4.6645112 \| -2.3170141 \| -2.5599525 \| \| C \| 6.0677122 \| -3.6882271 \| -1.6610945 \| \| H \| 6.6997662 \| -3.7377361 \| -2.5465805 \| \| C \| 6.3969022 \| -4.4232801 \| -0.5261225 \| \| H \| 7.2889512 \| -5.0477581 \| -0.5186775 \| \| C \| 5.5802342 \| -4.3566011 \| 0.5988595 \| \| H \| 5.8330952 \| -4.9265081 \| 1.4918955 \| \| C \| 4.4418802 \| -3.5578591 \| 0.5875045 \| \| H \| 3.8003792 \| -3.5001991 \| 1.4674425 \| \| N \| 0.2665612 \| 0.0197799 \| 1.9014975 \| \| C \| 1.4905272 \| 0.0473539 \| 2.5353695 \| \| H \| 2.4109952 \| 0.0715799 \| 1.9644515 \| \| C \| 1.3099592 \| 0.0378139 \| 3.8841165 \| \| H \| 2.0014362 \| 0.0512329 \| 4.7145975 \| \| H \| -0.5463858 \| -0.0082971 \| 4.9529935 \| \| C \| -0.6571078 \| -0.0059201 \| 2.8466745 \| \| C \| -2.1279448 \| -0.0384141 \| 2.6624885 \| \| H \| -2.6444848 \| -0.0336981 \| 3.6297975 \| \| H \| -2.4661058 \| 0.8291349 \| 2.0805005 \| \| H \| -2.4317638 \| -0.9341551 \| 2.1047395 \| |

| [Co(TPP)(2-MeHIm)] S = 1/2 | |
| --- | --- |
| \| B3LYP \| \| E= -3561.131874 Hartree \| \| \| --- \| --- \| --- \| --- \| \| Co \| 0.0703858 \| 0.0453538 \| -0.4097379 \| \| N \| -0.6513142 \| 1.9063718 \| -0.4592709 \| \| N \| -1.7829462 \| -0.6629142 \| -0.5531249 \| \| C \| 1.4478848 \| 3.1528538 \| -0.0800019 \| \| C \| -3.0477072 \| 1.4409628 \| -0.8455519 \| \| C \| 0.0605208 \| 3.0655698 \| -0.2406539 \| \| C \| -1.9580912 \| 2.2994968 \| -0.6608829 \| \| C \| -2.9461692 \| 0.0478568 \| -0.7513689 \| \| C \| -2.1676222 \| -1.9725272 \| -0.3678939 \| \| C \| -0.8234312 \| 4.2083558 \| -0.2791139 \| \| H \| -0.5207482 \| 5.2333738 \| -0.1237359 \| \| C \| -2.0630152 \| 3.7387598 \| -0.5789219 \| \| H \| -2.9723142 \| 4.3043108 \| -0.7197699 \| \| C \| -4.0864732 \| -0.8374712 \| -0.6979469 \| \| H \| -5.1147522 \| -0.5315242 \| -0.8247429 \| \| C \| -3.6065752 \| -2.0839022 \| -0.4395829 \| \| H \| -4.1624792 \| -3.0041732 \| -0.3334489 \| \| C \| 2.0602868 \| 4.4842908 \| 0.2159201 \| \| C \| 2.0750398 \| 5.5092688 \| -0.7423159 \| \| H \| 1.6254028 \| 5.3284478 \| -1.7145899 \| \| C \| 2.6633478 \| 6.7423748 \| -0.4570389 \| \| H \| 2.6688038 \| 7.5242918 \| -1.2119789 \| \| C \| 3.2506148 \| 6.9681138 \| 0.7901491 \| \| H \| 3.7101868 \| 7.9276898 \| 1.0117471 \| \| C \| 3.2464488 \| 5.9531628 \| 1.7497251 \| \| H \| 3.7012228 \| 6.1206478 \| 2.7226581 \| \| C \| 2.6563388 \| 4.7210058 \| 1.4639681 \| \| H \| 2.6524718 \| 3.9287308 \| 2.2075011 \| \| C \| -4.4027592 \| 2.0294798 \| -1.0716229 \| \| C \| -5.0525742 \| 1.8581788 \| -2.3034369 \| \| H \| -4.5498972 \| 1.3041848 \| -3.0912439 \| \| C \| -6.3246422 \| 2.3910428 \| -2.5179979 \| \| H \| -6.8117202 \| 2.2524598 \| -3.4796809 \| \| C \| -6.9680462 \| 3.1027038 \| -1.5028929 \| \| H \| -7.9583752 \| 3.5178578 \| -1.6699859 \| \| C \| -6.3323592 \| 3.2768838 \| -0.2712889 \| \| H \| -6.8282822 \| 3.8244808 \| 0.5259831 \| \| C \| -5.0611732 \| 2.7422698 \| -0.0576349 \| \| H \| -4.5671642 \| 2.8721888 \| 0.9013421 \| \| N \| -0.3102072 \| -0.6051172 \| 3.9038871 \| \| N \| 0.7913288 \| -1.8131232 \| -0.5623549 \| \| N \| 1.9269148 \| 0.7605578 \| -0.4815589 \| \| C \| -1.3081722 \| -3.0685232 \| -0.2267989 \| \| C \| 3.1975828 \| -1.3305812 \| -0.8399679 \| \| C \| 0.0788298 \| -2.9785662 \| -0.3847199 \| \| C \| 2.1028398 \| -2.1963862 \| -0.7344109 \| \| C \| 3.0940878 \| 0.0564338 \| -0.6833659 \| \| C \| 2.3086078 \| 2.0634458 \| -0.2525889 \| \| C \| 0.9659268 \| -4.1185932 \| -0.4392799 \| \| H \| 0.6631338 \| -5.1482902 \| -0.3187769 \| \| C \| 2.2104018 \| -3.6376992 \| -0.6970539 \| \| H \| 3.1252778 \| -4.1960042 \| -0.8304749 \| \| C \| 4.2335108 \| 0.9420428 \| -0.5923389 \| \| H \| 5.2634018 \| 0.6399038 \| -0.7151799 \| \| C \| 3.7486958 \| 2.1795938 \| -0.3098709 \| \| H \| 4.3012338 \| 3.0974208 \| -0.1709579 \| \| C \| -1.9231942 \| -4.4041612 \| 0.0401941 \| \| C \| -1.9320042 \| -5.4138792 \| -0.9341019 \| \| H \| -1.4754542 \| -5.2179852 \| -1.9001499 \| \| C \| -2.5232502 \| -6.6508402 \| -0.6724229 \| \| H \| -2.5238652 \| -7.4206602 \| -1.4396739 \| \| C \| -3.1198032 \| -6.8960952 \| 0.5666211 \| \| H \| -3.5814692 \| -7.8587392 \| 0.7696131 \| \| C \| -3.1218852 \| -5.8965552 \| 1.5423141 \| \| H \| -3.5835052 \| -6.0794782 \| 2.5092641 \| \| C \| -2.5286542 \| -4.6609462 \| 1.2798441 \| \| H \| -2.5288152 \| -3.8801282 \| 2.0357991 \| \| C \| 4.5581808 \| -1.9116632 \| -1.0563279 \| \| C \| 5.2291388 \| -1.7096102 \| -2.2718539 \| \| H \| 4.7406568 \| -1.1354072 \| -3.0541439 \| \| C \| 6.5045418 \| -2.2381302 \| -2.4776539 \| \| H \| 7.0086628 \| -2.0752772 \| -3.4266709 \| \| C \| 7.1294708 \| -2.9762742 \| -1.4699639 \| \| H \| 8.1224248 \| -3.3878772 \| -1.6301969 \| \| C \| 6.4721118 \| -3.1815462 \| -0.2546679 \| \| H \| 6.9535068 \| -3.7501722 \| 0.5367461 \| \| C \| 5.1976398 \| -2.6511942 \| -0.0496829 \| \| H \| 4.6861038 \| -2.8060012 \| 0.8963251 \| \| N \| 0.1544668 \| -0.1580652 \| 1.8017861 \| \| C \| 1.2900328 \| -0.6512312 \| 2.4135151 \| \| H \| 2.2062398 \| -0.7690042 \| 1.8555431 \| \| C \| 1.0248198 \| -0.9356912 \| 3.7243411 \| \| H \| 1.6330508 \| -1.3234332 \| 4.5269621 \| \| H \| -0.8328532 \| -0.6816132 \| 4.7649681 \| \| C \| -0.8011142 \| -0.1380362 \| 2.7155401 \| \| C \| -2.2167722 \| 0.2870288 \| 2.5125441 \| \| H \| -2.6785542 \| 0.5913588 \| 3.4584701 \| \| H \| -2.2476602 \| 1.1261168 \| 1.8155261 \| \| H \| -2.8139152 \| -0.5218312 \| 2.0758691 \| | \| M06 \| \| E= -3559.302616 Hartree \| \| \| --- \| --- \| --- \| --- \| \| Co \| -0.0550903 \| 0.0402764 \| -0.4353833 \| \| N \| 0.5445337 \| -1.8435616 \| -0.5539973 \| \| N \| 1.8279927 \| 0.6290654 \| -0.5268693 \| \| C \| -1.6162663 \| -2.9589946 \| -0.1930013 \| \| C \| 2.9566677 \| -1.5235466 \| -0.9123793 \| \| C \| -0.2289983 \| -2.9552866 \| -0.3437253 \| \| C \| 1.8181837 \| -2.3131126 \| -0.7483913 \| \| C \| 2.9398237 \| -0.1353386 \| -0.7710593 \| \| C \| 2.2955607 \| 1.8973544 \| -0.3008743 \| \| C \| 0.5787577 \| -4.1447636 \| -0.3806523 \| \| H \| 0.2124617 \| -5.1528586 \| -0.2268043 \| \| C \| 1.8418217 \| -3.7495526 \| -0.6751503 \| \| H \| 2.7208207 \| -4.3675556 \| -0.8161983 \| \| C \| 4.1285397 \| 0.6711454 \| -0.7107713 \| \| H \| 5.1372717 \| 0.3079384 \| -0.8706033 \| \| C \| 3.7305757 \| 1.9296214 \| -0.3980953 \| \| H \| 4.3436737 \| 2.8147674 \| -0.2728713 \| \| C \| -2.3088563 \| -4.2368116 \| 0.1169057 \| \| C \| -2.3678613 \| -5.2813196 \| -0.8105813 \| \| H \| -1.8915813 \| -5.1522486 \| -1.7826583 \| \| C \| -3.0324733 \| -6.4642176 \| -0.5047273 \| \| H \| -3.0739503 \| -7.2663736 \| -1.2401093 \| \| C \| -3.6518393 \| -6.6184736 \| 0.7319577 \| \| H \| -4.1740033 \| -7.5437316 \| 0.9706457 \| \| C \| -3.6047713 \| -5.5828766 \| 1.6608507 \| \| H \| -4.0868093 \| -5.6969886 \| 2.6308617 \| \| C \| -2.9391033 \| -4.4010726 \| 1.3545577 \| \| H \| -2.8967963 \| -3.5842766 \| 2.0764797 \| \| C \| 4.2676407 \| -2.1844336 \| -1.1496283 \| \| C \| 4.9055257 \| -2.0619896 \| -2.3873793 \| \| H \| 4.4153337 \| -1.4946526 \| -3.1786313 \| \| C \| 6.1454807 \| -2.6526746 \| -2.6075693 \| \| H \| 6.6274907 \| -2.5504516 \| -3.5787783 \| \| C \| 6.7673827 \| -3.3719906 \| -1.5915843 \| \| H \| 7.7391517 \| -3.8325396 \| -1.7629163 \| \| C \| 6.1428477 \| -3.4988276 \| -0.3542613 \| \| H \| 6.6266667 \| -4.0544636 \| 0.4477807 \| \| C \| 4.9029557 \| -2.9077716 \| -0.1357253 \| \| H \| 4.4140367 \| -2.9996296 \| 0.8350907 \| \| N \| 0.1003507 \| -0.4001726 \| 3.9413257 \| \| N \| -0.6511683 \| 1.9280364 \| -0.4943433 \| \| N \| -1.9353613 \| -0.5482476 \| -0.5810503 \| \| C \| 1.5096297 \| 3.0329474 \| -0.0970703 \| \| C \| -3.0644553 \| 1.6205864 \| -0.8630323 \| \| C \| 0.1230367 \| 3.0327134 \| -0.2544943 \| \| C \| -1.9244343 \| 2.4029014 \| -0.6754003 \| \| C \| -3.0486293 \| 0.2270784 \| -0.7751353 \| \| C \| -2.4018193 \| -1.8202646 \| -0.3781793 \| \| C \| -0.6828093 \| 4.2244294 \| -0.2675673 \| \| H \| -0.3162733 \| 5.2284244 \| -0.0900433 \| \| C \| -1.9453163 \| 3.8378064 \| -0.5721973 \| \| H \| -2.8224263 \| 4.4607914 \| -0.7008813 \| \| C \| -4.2386783 \| -0.5797276 \| -0.7194313 \| \| H \| -5.2494983 \| -0.2095626 \| -0.8459843 \| \| C \| -3.8378563 \| -1.8475046 \| -0.4531913 \| \| H \| -4.4507813 \| -2.7343686 \| -0.3403343 \| \| C \| 2.2051977 \| 4.3104614 \| 0.2085147 \| \| C \| 2.2528097 \| 5.3531654 \| -0.7221513 \| \| H \| 1.7629897 \| 5.2223094 \| -1.6871703 \| \| C \| 2.9230777 \| 6.5358884 \| -0.4291703 \| \| H \| 2.9546017 \| 7.3360074 \| -1.1672343 \| \| C \| 3.5605147 \| 6.6927864 \| 0.7980467 \| \| H \| 4.0865787 \| 7.6182454 \| 1.0270637 \| \| C \| 3.5262297 \| 5.6593704 \| 1.7296097 \| \| H \| 4.0221177 \| 5.7749754 \| 2.6923747 \| \| C \| 2.8548107 \| 4.4776624 \| 1.4353987 \| \| H \| 2.8247167 \| 3.6633954 \| 2.1605267 \| \| C \| -4.3748373 \| 2.2892764 \| -1.0768183 \| \| C \| -5.0391523 \| 2.1632104 \| -2.3007883 \| \| H \| -4.5697343 \| 1.5867794 \| -3.0980773 \| \| C \| -6.2779193 \| 2.7630064 \| -2.5008143 \| \| H \| -6.7795703 \| 2.6581074 \| -3.4617423 \| \| C \| -6.8728973 \| 3.4959214 \| -1.4783303 \| \| H \| -7.8438933 \| 3.9636494 \| -1.6339953 \| \| C \| -6.2222983 \| 3.6264404 \| -0.2548713 \| \| H \| -6.6852443 \| 4.1920104 \| 0.5524987 \| \| C \| -4.9836073 \| 3.0263374 \| -0.0560793 \| \| H \| -4.4753913 \| 3.1190254 \| 0.9041397 \| \| N \| -0.2071763 \| -0.0391926 \| 1.7969657 \| \| C \| -1.4182383 \| 0.0429474 \| 2.4437867 \| \| H \| -2.3291253 \| 0.2559764 \| 1.8943857 \| \| C \| -1.2537083 \| -0.1780436 \| 3.7790077 \| \| H \| -1.9447693 \| -0.1981286 \| 4.6101867 \| \| H \| 0.5750427 \| -0.5983126 \| 4.8120367 \| \| C \| 0.6949867 \| -0.3074846 \| 2.7184837 \| \| C \| 2.1544007 \| -0.4835556 \| 2.5136207 \| \| H \| 2.6523407 \| -0.7767316 \| 3.4460717 \| \| H \| 2.3468217 \| -1.2549246 \| 1.7566667 \| \| H \| 2.6140207 \| 0.4457674 \| 2.1511187 \| |

| [Co(TPP)(2-MeIm^‒^)]^‒^  S = 3/2 | |
| --- | --- |
| \| B3LYP \| \| E= -3560.579658 Hartree \| \| \| --- \| --- \| --- \| --- \| \| Co \| 0.0442914 \| 0.0158495 \| -0.0413643 \| \| N \| 1.7867064 \| 1.1342455 \| -0.4126003 \| \| N \| -1.0782736 \| 1.7467355 \| -0.4368623 \| \| N \| -1.6805096 \| -1.0903695 \| -0.5010403 \| \| N \| 1.1797844 \| -1.7055855 \| -0.5269893 \| \| C \| 3.0652914 \| 0.6606325 \| -0.5431553 \| \| C \| 1.8601304 \| 2.5008955 \| -0.4091573 \| \| C \| -0.5946726 \| 3.0280415 \| -0.3751883 \| \| C \| -2.4442716 \| 1.8336155 \| -0.5163333 \| \| C \| -2.9612396 \| -0.6146885 \| -0.6085193 \| \| C \| -1.7589536 \| -2.4575915 \| -0.4687673 \| \| C \| 0.6956694 \| -2.9824805 \| -0.4187633 \| \| C \| 2.5461514 \| -1.7913405 \| -0.5218113 \| \| C \| 3.9883804 \| 1.7770575 \| -0.6511763 \| \| H \| 5.0563254 \| 1.6978135 \| -0.7973283 \| \| C \| 3.2446404 \| 2.9137545 \| -0.5609143 \| \| H \| 3.5919094 \| 3.9359225 \| -0.6166153 \| \| C \| -1.7034636 \| 3.9650085 \| -0.3892423 \| \| H \| -1.6188566 \| 5.0408985 \| -0.3321543 \| \| C \| -2.8440996 \| 3.2279265 \| -0.4947513 \| \| H \| -3.8606706 \| 3.5907165 \| -0.5526633 \| \| C \| -3.8919086 \| -1.7277115 \| -0.6492163 \| \| H \| -4.9668996 \| -1.6450885 \| -0.7258143 \| \| C \| -3.1489256 \| -2.8662125 \| -0.5664713 \| \| H \| -3.5036046 \| -3.8872175 \| -0.5774783 \| \| C \| 1.8037904 \| -3.9166665 \| -0.3252403 \| \| H \| 1.7153834 \| -4.9866395 \| -0.2011993 \| \| C \| 2.9473224 \| -3.1815715 \| -0.3959973 \| \| H \| 3.9654714 \| -3.5396545 \| -0.3393323 \| \| C \| 3.4394974 \| -0.6995365 \| -0.5663913 \| \| C \| 0.7677144 \| 3.3914235 \| -0.3377873 \| \| C \| -3.3335026 \| 0.7441355 \| -0.6064693 \| \| C \| -0.6666376 \| -3.3468065 \| -0.3873763 \| \| C \| 4.9000224 \| -1.0098245 \| -0.6105463 \| \| C \| 5.4575314 \| -1.7112285 \| -1.6923013 \| \| H \| 4.8063024 \| -2.0229795 \| -2.5039473 \| \| C \| 6.8215084 \| -2.0039125 \| -1.7313843 \| \| H \| 7.2325404 \| -2.5448535 \| -2.5806633 \| \| C \| 7.6571984 \| -1.5984875 \| -0.6879143 \| \| H \| 8.7202004 \| -1.8257015 \| -0.7174693 \| \| C \| 7.1163624 \| -0.9005335 \| 0.3948087 \| \| H \| 7.7562404 \| -0.5867105 \| 1.2161277 \| \| C \| 5.7518784 \| -0.6110555 \| 0.4332147 \| \| H \| 5.3281064 \| -0.0752615 \| 1.2776047 \| \| C \| 1.0940114 \| 4.8479445 \| -0.2465773 \| \| C \| 0.7857314 \| 5.7340515 \| -1.2911873 \| \| H \| 0.2968694 \| 5.3452175 \| -2.1799593 \| \| C \| 1.1017124 \| 7.0903655 \| -1.1969493 \| \| H \| 0.8570124 \| 7.7593665 \| -2.0188013 \| \| C \| 1.7369864 \| 7.5856815 \| -0.0555833 \| \| H \| 1.9843534 \| 8.6420275 \| 0.0184247 \| \| C \| 2.0528924 \| 6.7140275 \| 0.9895377 \| \| H \| 2.5442984 \| 7.0899475 \| 1.8837737 \| \| C \| 1.7342484 \| 5.3585965 \| 0.8943557 \| \| H \| 1.9751224 \| 4.6779965 \| 1.7059427 \| \| C \| -4.7959506 \| 1.0537735 \| -0.6488673 \| \| C \| -5.5566246 \| 0.8129245 \| -1.8033503 \| \| H \| -5.0631226 \| 0.3999885 \| -2.6787923 \| \| C \| -6.9233876 \| 1.0963975 \| -1.8320853 \| \| H \| -7.4951606 \| 0.9047405 \| -2.7371883 \| \| C \| -7.5534396 \| 1.6293995 \| -0.7050423 \| \| H \| -8.6177596 \| 1.8513965 \| -0.7264933 \| \| C \| -6.8070786 \| 1.8736435 \| 0.4506047 \| \| H \| -7.2894996 \| 2.2824365 \| 1.3352807 \| \| C \| -5.4416246 \| 1.5865055 \| 0.4785867 \| \| H \| -4.8588436 \| 1.7661115 \| 1.3774947 \| \| C \| -0.9880086 \| -4.8015685 \| -0.2625953 \| \| C \| -0.6603396 \| -5.7107575 \| -1.2809773 \| \| H \| -0.1635856 \| -5.3402335 \| -2.1732613 \| \| C \| -0.9663016 \| -7.0669195 \| -1.1561533 \| \| H \| -0.7068496 \| -7.7543975 \| -1.9580223 \| \| C \| -1.6091036 \| -7.5387685 \| -0.0091023 \| \| H \| -1.8482006 \| -8.5950605 \| 0.0891177 \| \| C \| -1.9414656 \| -6.6442655 \| 1.0114217 \| \| H \| -2.4362716 \| -7.0022245 \| 1.9110737 \| \| C \| -1.6332866 \| -5.2889825 \| 0.8857587 \| \| H \| -1.8840716 \| -4.5912165 \| 1.6794877 \| \| N \| 0.1872154 \| -0.1281015 \| 1.9779887 \| \| N \| -0.2309176 \| -0.2768215 \| 4.1984347 \| \| C \| -0.7413646 \| -0.0810015 \| 2.9767447 \| \| C \| 1.1186024 \| -0.4659555 \| 3.9749037 \| \| H \| 1.8031854 \| -0.6500385 \| 4.7954307 \| \| C \| 1.3817434 \| -0.3771695 \| 2.6264197 \| \| H \| 2.3041414 \| -0.4700025 \| 2.0691387 \| \| C \| -2.1991946 \| 0.1552935 \| 2.7211217 \| \| H \| -2.3615566 \| 1.0727305 \| 2.1425407 \| \| H \| -2.6511386 \| -0.6634095 \| 2.1467387 \| \| H \| -2.7238436 \| 0.2413695 \| 3.6773037 \| | \| M06 \| \| E= -3558.75809 Hartree \| \| \| --- \| --- \| --- \| --- \| \| Co \| -0.0414822 \| 0.0084001 \| -0.0726130 \| \| N \| -2.0689932 \| -0.3121369 \| -0.5153770 \| \| N \| 0.2953728 \| -1.9955789 \| -0.5279780 \| \| N \| 2.0003858 \| 0.3334911 \| -0.4828050 \| \| N \| -0.3497582 \| 2.0194941 \| -0.5523320 \| \| C \| -3.0402702 \| 0.6355771 \| -0.6031720 \| \| C \| -2.6909022 \| -1.5201629 \| -0.5602270 \| \| C \| -0.6599762 \| -2.9721019 \| -0.4903700 \| \| C \| 1.5043238 \| -2.6287819 \| -0.5093200 \| \| C \| 2.9752458 \| -0.6152169 \| -0.5663540 \| \| C \| 2.6294778 \| 1.5417401 \| -0.4924740 \| \| C \| 0.5999238 \| 2.9931091 \| -0.4274880 \| \| C \| -1.5633772 \| 2.6462811 \| -0.4922530 \| \| C \| -4.3349662 \| 0.0035641 \| -0.7379350 \| \| H \| -5.2815412 \| 0.5173361 \| -0.8650680 \| \| C \| -4.1181592 \| -1.3363659 \| -0.7058930 \| \| H \| -4.8516012 \| -2.1301459 \| -0.7998010 \| \| C \| -0.0319282 \| -4.2695129 \| -0.4475000 \| \| H \| -0.5507012 \| -5.2204079 \| -0.3978560 \| \| C \| 1.3097038 \| -4.0576839 \| -0.4752400 \| \| H \| 2.0982878 \| -4.8021189 \| -0.4690260 \| \| C \| 4.2751758 \| 0.0139561 \| -0.6257580 \| \| H \| 5.2261708 \| -0.5031619 \| -0.6922680 \| \| C \| 4.0604338 \| 1.3533361 \| -0.5902290 \| \| H \| 4.8006528 \| 2.1444771 \| -0.6392150 \| \| C \| -0.0329892 \| 4.2802381 \| -0.2772530 \| \| H \| 0.4801858 \| 5.2233861 \| -0.1266200 \| \| C \| -1.3734062 \| 4.0656821 \| -0.3192780 \| \| H \| -2.1641302 \| 4.7994891 \| -0.2093320 \| \| C \| -2.8233682 \| 2.0231041 \| -0.5480550 \| \| C \| -2.0500932 \| -2.7683669 \| -0.4964350 \| \| C \| 2.7625958 \| -2.0019229 \| -0.5443340 \| \| C \| 1.9928358 \| 2.7915361 \| -0.4178620 \| \| C \| -4.0241522 \| 2.8972201 \| -0.5112100 \| \| C \| -4.2826722 \| 3.8215941 \| -1.5295200 \| \| H \| -3.5831762 \| 3.8916421 \| -2.3627140 \| \| C \| -5.4097192 \| 4.6354801 \| -1.4868050 \| \| H \| -5.5938812 \| 5.3463631 \| -2.2919480 \| \| C \| -6.3018862 \| 4.5390391 \| -0.4226890 \| \| H \| -7.1856702 \| 5.1753821 \| -0.3887270 \| \| C \| -6.0558112 \| 3.6256791 \| 0.5986290 \| \| H \| -6.7428152 \| 3.5497471 \| 1.4408700 \| \| C \| -4.9273492 \| 2.8146561 \| 0.5555160 \| \| H \| -4.7228722 \| 2.1077471 \| 1.3600640 \| \| C \| -2.9263792 \| -3.9678709 \| -0.4251290 \| \| C \| -3.0058092 \| -4.8800709 \| -1.4818420 \| \| H \| -2.4045452 \| -4.7022339 \| -2.3736380 \| \| C \| -3.8397952 \| -5.9908489 \| -1.4038040 \| \| H \| -3.8915852 \| -6.6881169 \| -2.2396330 \| \| C \| -4.6105632 \| -6.2073079 \| -0.2650610 \| \| H \| -5.2641382 \| -7.0768749 \| -0.2030700 \| \| C \| -4.5413112 \| -5.3054499 \| 0.7932190 \| \| H \| -5.1369742 \| -5.4696559 \| 1.6905910 \| \| C \| -3.7073002 \| -4.1955409 \| 0.7134390 \| \| H \| -3.6414462 \| -3.4875959 \| 1.5401310 \| \| C \| 3.9635648 \| -2.8803199 \| -0.5228210 \| \| C \| 4.7877718 \| -3.0103459 \| -1.6449820 \| \| H \| 4.5343498 \| -2.4545869 \| -2.5481610 \| \| C \| 5.9105808 \| -3.8311829 \| -1.6138720 \| \| H \| 6.5397268 \| -3.9197809 \| -2.4991910 \| \| C \| 6.2255448 \| -4.5417879 \| -0.4593050 \| \| H \| 7.1035208 \| -5.1865449 \| -0.4350340 \| \| C \| 5.4124198 \| -4.4218589 \| 0.6643050 \| \| H \| 5.6537838 \| -4.9699659 \| 1.5744830 \| \| C \| 4.2937098 \| -3.5962329 \| 0.6328900 \| \| H \| 3.6568048 \| -3.4931219 \| 1.5119770 \| \| C \| 2.8632028 \| 3.9923881 \| -0.3217610 \| \| C \| 2.8718168 \| 4.9647551 \| -1.3281450 \| \| H \| 2.2220158 \| 4.8283391 \| -2.1925900 \| \| C \| 3.6948368 \| 6.0821051 \| -1.2360050 \| \| H \| 3.6894268 \| 6.8246471 \| -2.0335480 \| \| C \| 4.5280538 \| 6.2476741 \| -0.1332800 \| \| H \| 5.1739998 \| 7.1220551 \| -0.0606010 \| \| C \| 4.5296388 \| 5.2886161 \| 0.8756560 \| \| H \| 5.1731708 \| 5.4118441 \| 1.7460770 \| \| C \| 3.7050168 \| 4.1728301 \| 0.7821690 \| \| H \| 3.6969738 \| 3.4234491 \| 1.5739310 \| \| N \| -0.2966042 \| -0.0212479 \| 1.9307130 \| \| N \| 0.0586758 \| -0.1116969 \| 4.1620530 \| \| C \| 0.6043988 \| -0.0564509 \| 2.9480050 \| \| C \| -1.2924142 \| -0.1109499 \| 3.9171900 \| \| H \| -2.0125712 \| -0.1485609 \| 4.7308520 \| \| C \| -1.5203842 \| -0.0603389 \| 2.5653180 \| \| H \| -2.4487432 \| -0.0417999 \| 2.0004130 \| \| C \| 2.0800568 \| -0.0492069 \| 2.7346840 \| \| H \| 2.4155168 \| -0.9104129 \| 2.1356260 \| \| H \| 2.4202358 \| 0.8537341 \| 2.2039900 \| \| H \| 2.5786248 \| -0.0895579 \| 3.7100170 \| |

| [Co(TPP)(2-MeIm^‒^)]^‒^  S = 1/2 | |
| --- | --- |
| \| B3LYP \| \| E= -3560.575873 Hartree \| \| \| --- \| --- \| --- \| --- \| \| Co \| 0.0529893 \| 0.1088682 \| -0.4109852 \| \| N \| 1.6705373 \| 1.2639172 \| -0.4153332 \| \| N \| -1.1184007 \| 1.7223562 \| -0.3967732 \| \| N \| -1.5483907 \| -1.0222148 \| -0.7012982 \| \| N \| 1.2161163 \| -1.4806888 \| -0.7138552 \| \| C \| 2.9679483 \| 0.9060712 \| -0.7101752 \| \| C \| 1.7022983 \| 2.6024022 \| -0.1103112 \| \| C \| -0.7321187 \| 2.9960572 \| -0.0532052 \| \| C \| -2.4737667 \| 1.7840422 \| -0.6365252 \| \| C \| -2.8432927 \| -0.6326568 \| -0.9387492 \| \| C \| -1.5904297 \| -2.3768698 \| -0.4901592 \| \| C \| 0.8317853 \| -2.7714668 \| -0.4447582 \| \| C \| 2.5730363 \| -1.5189418 \| -0.8966752 \| \| C \| 3.8300023 \| 2.0663822 \| -0.6275962 \| \| H \| 4.8878743 \| 2.0740612 \| -0.8471322 \| \| C \| 3.0538423 \| 3.1069142 \| -0.2235552 \| \| H \| 3.3493843 \| 4.1327452 \| -0.0550052 \| \| C \| -1.8800677 \| 3.8756922 \| -0.0377642 \| \| H \| -1.8601247 \| 4.9237272 \| 0.2236318 \| \| C \| -2.9479707 \| 3.1365012 \| -0.4420062 \| \| H \| -3.9679967 \| 3.4655242 \| -0.5786262 \| \| C \| -3.7184847 \| -1.7863408 \| -0.9314452 \| \| H \| -4.7864877 \| -1.7567948 \| -1.0940302 \| \| C \| -2.9457117 \| -2.8610008 \| -0.6117552 \| \| H \| -3.2450277 \| -3.8922498 \| -0.4854192 \| \| C \| 1.9785213 \| -3.6501128 \| -0.4632902 \| \| H \| 1.9444403 \| -4.7126658 \| -0.2674972 \| \| C \| 3.0535283 \| -2.8824628 \| -0.7868842 \| \| H \| 4.0852743 \| -3.1878408 \| -0.8881772 \| \| C \| 3.4124733 \| -0.3969268 \| -0.9727662 \| \| C \| 0.5899783 \| 3.4146032 \| 0.1478578 \| \| C \| -3.2945907 \| 0.6940102 \| -0.9563422 \| \| C \| -0.4830097 \| -3.1946388 \| -0.2462552 \| \| C \| 4.8619943 \| -0.6323848 \| -1.2364702 \| \| C \| 5.2796663 \| -1.1848568 \| -2.4581952 \| \| H \| 4.5298743 \| -1.4207058 \| -3.2081342 \| \| C \| 6.6303633 \| -1.4291718 \| -2.7105922 \| \| H \| 6.9321333 \| -1.8542958 \| -3.6650352 \| \| C \| 7.5915823 \| -1.1257168 \| -1.7434982 \| \| H \| 8.6443093 \| -1.3155698 \| -1.9388202 \| \| C \| 7.1895493 \| -0.5815168 \| -0.5210942 \| \| H \| 7.9282263 \| -0.3529958 \| 0.2435358 \| \| C \| 5.8382913 \| -0.3409738 \| -0.2693792 \| \| H \| 5.5221453 \| 0.0675222 \| 0.6858718 \| \| C \| 0.8507423 \| 4.8209192 \| 0.5778038 \| \| C \| 0.5604873 \| 5.9147352 \| -0.2531402 \| \| H \| 0.1271483 \| 5.7293362 \| -1.2318672 \| \| C \| 0.8284923 \| 7.2200732 \| 0.1624138 \| \| H \| 0.5991323 \| 8.0537692 \| -0.4973022 \| \| C \| 1.3985813 \| 7.4548512 \| 1.4160158 \| \| H \| 1.6089043 \| 8.4714182 \| 1.7398908 \| \| C \| 1.6990573 \| 6.3745032 \| 2.2493298 \| \| H \| 2.1420743 \| 6.5465812 \| 3.2273478 \| \| C \| 1.4280613 \| 5.0700532 \| 1.8334448 \| \| H \| 1.6574263 \| 4.2265302 \| 2.4782478 \| \| C \| -4.7463767 \| 0.9283882 \| -1.2136632 \| \| C \| -5.2959847 \| 0.6582582 \| -2.4767022 \| \| H \| -4.6423257 \| 0.2927712 \| -3.2637882 \| \| C \| -6.6562177 \| 0.8526132 \| -2.7226202 \| \| H \| -7.0616617 \| 0.6400982 \| -3.7091362 \| \| C \| -7.4930057 \| 1.3216822 \| -1.7072852 \| \| H \| -8.5527447 \| 1.4739742 \| -1.8975272 \| \| C \| -6.9594537 \| 1.5891852 \| -0.4439622 \| \| H \| -7.6044017 \| 1.9439792 \| 0.3564448 \| \| C \| -5.6000887 \| 1.3904012 \| -0.1988072 \| \| H \| -5.1850177 \| 1.5822512 \| 0.7863288 \| \| C \| -0.7272567 \| -4.5705588 \| 0.2825778 \| \| C \| -0.7589367 \| -5.7014788 \| -0.5430492 \| \| H \| -0.6005877 \| -5.5812758 \| -1.6118082 \| \| C \| -0.9926187 \| -6.9677418 \| -0.0000912 \| \| H \| -1.0169907 \| -7.8394908 \| -0.6503792 \| \| C \| -1.1963207 \| -7.1107868 \| 1.3743578 \| \| H \| -1.3778857 \| -8.0960108 \| 1.7984048 \| \| C \| -1.1641097 \| -5.9847848 \| 2.2022918 \| \| H \| -1.3167437 \| -6.0895928 \| 3.2736138 \| \| C \| -0.9320097 \| -4.7173918 \| 1.6648228 \| \| H \| -0.8929137 \| -3.8373218 \| 2.3062148 \| \| N \| 0.1378213 \| -0.3342618 \| 1.7236458 \| \| N \| -0.3090577 \| -1.6137228 \| 3.5485948 \| \| C \| -0.8056487 \| -0.8592288 \| 2.5479758 \| \| C \| 1.0537423 \| -1.5649528 \| 3.3530438 \| \| H \| 1.7377283 \| -2.0793308 \| 4.0198808 \| \| C \| 1.3311983 \| -0.7825318 \| 2.2484988 \| \| H \| 2.2738083 \| -0.5306008 \| 1.7795098 \| \| C \| -2.2761007 \| -0.6166608 \| 2.3716378 \| \| H \| -2.4451127 \| 0.2965112 \| 1.7940878 \| \| H \| -2.7744757 \| -1.4393638 \| 1.8408698 \| \| H \| -2.7626507 \| -0.5151338 \| 3.3485078 \| | \| M06 \| \| E= -3558.751403 Hartree \| \| \| --- \| --- \| --- \| --- \| \| Co \| 0.0660245 \| 0.0265339 \| -0.4353049 \| \| N \| 1.7113255 \| 1.1222199 \| -0.5402219 \| \| N \| -1.0366165 \| 1.6733709 \| -0.5537079 \| \| N \| -1.5698815 \| -1.0642481 \| -0.6241139 \| \| N \| 1.1646825 \| -1.6196591 \| -0.5991349 \| \| C \| 2.9952705 \| 0.7008899 \| -0.7570849 \| \| C \| 1.7944915 \| 2.4685519 \| -0.3252339 \| \| C \| -0.6132685 \| 2.9449909 \| -0.2814019 \| \| C \| -2.3889175 \| 1.7669119 \| -0.7434699 \| \| C \| -2.8473665 \| -0.6386721 \| -0.8663489 \| \| C \| -1.6654385 \| -2.4084561 \| -0.3990419 \| \| C \| 0.7375905 \| -2.8873951 \| -0.3121389 \| \| C \| 2.5218135 \| -1.7077321 \| -0.7260349 \| \| C \| 3.9012845 \| 1.8210209 \| -0.7418269 \| \| H \| 4.9676345 \| 1.7678879 \| -0.9298739 \| \| C \| 3.1589075 \| 2.9148399 \| -0.4424369 \| \| H \| 3.4900655 \| 3.9426729 \| -0.3431609 \| \| C \| -1.7282245 \| 3.8560629 \| -0.2703949 \| \| H \| -1.6673145 \| 4.9166169 \| -0.0543639 \| \| C \| -2.8258775 \| 3.1303669 \| -0.5983129 \| \| H \| -3.8481965 \| 3.4750189 \| -0.7075979 \| \| C \| -3.7642575 \| -1.7483981 \| -0.8354379 \| \| H \| -4.8333565 \| -1.6798371 \| -1.0035619 \| \| C \| -3.0317935 \| -2.8454221 \| -0.5203399 \| \| H \| -3.3719125 \| -3.8681221 \| -0.4000589 \| \| C \| 1.8553195 \| -3.7909341 \| -0.2347669 \| \| H \| 1.7915625 \| -4.8437601 \| 0.0154521 \| \| C \| 2.9618145 \| -3.0648401 \| -0.5270249 \| \| H \| 3.9931765 \| -3.3981731 \| -0.5566689 \| \| C \| 3.3985585 \| -0.6299511 \| -0.8758469 \| \| C \| 0.7176505 \| 3.3334059 \| -0.1177169 \| \| C \| -3.2525725 \| 0.6928389 \| -0.9541209 \| \| C \| -0.5942605 \| -3.2733281 \| -0.1629889 \| \| C \| 4.8428315 \| -0.9313901 \| -1.0483079 \| \| C \| 5.3055315 \| -1.5409701 \| -2.2188289 \| \| H \| 4.5877505 \| -1.7741441 \| -3.0054589 \| \| C \| 6.6533575 \| -1.8445801 \| -2.3798679 \| \| H \| 6.9961565 \| -2.3145601 \| -3.3012909 \| \| C \| 7.5619945 \| -1.5464651 \| -1.3686069 \| \| H \| 8.6180165 \| -1.7841431 \| -1.4930699 \| \| C \| 7.1129915 \| -0.9467921 \| -0.1950579 \| \| H \| 7.8155395 \| -0.7212611 \| 0.6065741 \| \| C \| 5.7649885 \| -0.6439931 \| -0.0360249 \| \| H \| 5.4034015 \| -0.1883261 \| 0.8861861 \| \| C \| 1.0229745 \| 4.7430409 \| 0.2360651 \| \| C \| 0.7518655 \| 5.7998569 \| -0.6392659 \| \| H \| 0.3024145 \| 5.5793729 \| -1.6077199 \| \| C \| 1.0570965 \| 7.1111129 \| -0.2887269 \| \| H \| 0.8419295 \| 7.9206779 \| -0.9857729 \| \| C \| 1.6443085 \| 7.3872729 \| 0.9426841 \| \| H \| 1.8839915 \| 8.4141499 \| 1.2171361 \| \| C \| 1.9253395 \| 6.3432359 \| 1.8195651 \| \| H \| 2.3823875 \| 6.5489259 \| 2.7869091 \| \| C \| 1.6182315 \| 5.0331259 \| 1.4689921 \| \| H \| 1.8279855 \| 4.2107249 \| 2.1528731 \| \| C \| -4.7054035 \| 0.9640019 \| -1.1178189 \| \| C \| -5.3344255 \| 0.8085059 \| -2.3554299 \| \| H \| -4.7354375 \| 0.5031579 \| -3.2137239 \| \| C \| -6.7012245 \| 1.0341359 \| -2.4907389 \| \| H \| -7.1764705 \| 0.9091969 \| -3.4634439 \| \| C \| -7.4581825 \| 1.4182879 \| -1.3880889 \| \| H \| -8.5283965 \| 1.5938119 \| -1.4933099 \| \| C \| -6.8418555 \| 1.5727369 \| -0.1489379 \| \| H \| -7.4291185 \| 1.8645549 \| 0.7211411 \| \| C \| -5.4766965 \| 1.3442369 \| -0.0148929 \| \| H \| -4.9866205 \| 1.4488189 \| 0.9540531 \| \| C \| -0.9080565 \| -4.6700251 \| 0.2283651 \| \| C \| -0.6251635 \| -5.7551071 \| -0.6069349 \| \| H \| -0.1590945 \| -5.5679771 \| -1.5746539 \| \| C \| -0.9405285 \| -7.0529461 \| -0.2174459 \| \| H \| -0.7159455 \| -7.8868811 \| -0.8820649 \| \| C \| -1.5478615 \| -7.2849401 \| 1.0132501 \| \| H \| -1.7952885 \| -8.3012951 \| 1.3184001 \| \| C \| -1.8377945 \| -6.2117171 \| 1.8514601 \| \| H \| -2.3078205 \| -6.3840131 \| 2.8190381 \| \| C \| -1.5210095 \| -4.9149461 \| 1.4625651 \| \| H \| -1.7342205 \| -4.0677341 \| 2.1151741 \| \| N \| 0.1539075 \| -0.0684281 \| 1.7053821 \| \| N \| -0.3021285 \| -0.3049551 \| 3.9136871 \| \| C \| -0.7771965 \| -0.0508351 \| 2.6854981 \| \| C \| 1.0358935 \| -0.5038851 \| 3.7060871 \| \| H \| 1.7084045 \| -0.7335741 \| 4.5300211 \| \| C \| 1.3213205 \| -0.3613641 \| 2.3658391 \| \| H \| 2.2668435 \| -0.4481721 \| 1.8324691 \| \| C \| -2.2235195 \| 0.2340689 \| 2.4462811 \| \| H \| -2.3711855 \| 1.1806709 \| 1.9018981 \| \| H \| -2.7206325 \| -0.5548421 \| 1.8577931 \| \| H \| -2.7340955 \| 0.3073059 \| 3.4143881 \| |
