# Supplementary material for: Proton mediated spin state transition of cobalt heme analogs
Source: Nat Commun. 2019 May 24;10:2303. doi: 10.1038/s41467-019-10357-z (PMC6534676; doi:10.1038/s41467-019-10357-z)
Supplement: Supplementary file 3 — Description of Additional Supplementary Files [file 41467_2019_10357_MOESM3_ESM.docx]

Description of Additional Supplementary Files

**Supplementary Data 1 :** Calculated Cartesian Coordinates and Electronic Energy of [Co(TPP)(2-MeHIm)] and [Co(TPP)(2-MeIm‒)]‒ at UM06 and UB3LYP level of theory.
